# Comparison of spatial transcriptomics technologies using tumor cryosections

**DOI:** 10.1186/s13059-025-03624-4

**Published:** 2025-06-20

**Authors:** Anne Rademacher, Alik Huseynov, Michele Bortolomeazzi, Sina Jasmin Wille, Sabrina Schumacher, Pooja Sant, Denise Keitel, Konstantin Okonechnikov, David R. Ghasemi, Kristian W. Pajtler, Jan-Philipp Mallm, Karsten Rippe

**Affiliations:** 1 Division of Chromatin Networks, German Cancer Research Center (DKFZ), Heidelberg, Germany; 2 Center for Quantitative Analysis of Molecular and Cellular Biosystems (BioQuant), Heidelberg University, Heidelberg, Germany; 3 German Cancer Research Center (DKFZ), Single Cell Open Lab, Heidelberg, Germany; 4 Hopp Children’s Cancer Center (KiTZ), Heidelberg, Germany; 5 National Center for Tumor Diseases (NCT), NCT Heidelberg, a partnership between DKFZ and Heidelberg University Hospital, Heidelberg, Germany; 6 Division of Pediatric Neurooncology, German Cancer Research Center (DKFZ) and German Cancer Consortium (DKTK), Heidelberg, Germany; 7 Heidelberg University, Heidelberg Faculty of Medicine, Department of Pediatric Oncology, Hematology, and Immunology, University Hospital Heidelberg, Heidelberg, Germany; 8 Present address: Department of Pediatric Hematology and Oncology, University Medical Center Hamburg-Eppendorf and Research Institute Children’s Cancer Center Hamburg, Hamburg, Germany

**Keywords:** Spatial transcriptomics, Visium, RNAscope, Molecular Cartography, Merscope, MERFISH, Xenium, Medulloblastoma

## Abstract

**Background:**

Spatial transcriptomics technologies are revolutionizing our understanding of intra-tumor heterogeneity and the tumor microenvironment by revealing single-cell molecular profiles within their spatial tissue context. The rapid development of spatial transcriptomics methods, each with unique characteristics, makes it challenging to select the most suitable technology for specific research objectives. Here, we compare four imaging-based approaches—RNAscope HiPlex, Molecular Cartography, Merscope, and Xenium—alongside Visium, a sequencing-based method. These technologies were employed to study cryosections of medulloblastoma with extensive nodularity (MBEN), a tumor chosen for its distinct microanatomical features.

**Results:**

Our analysis reveals that automated imaging-based spatial transcriptomics methods are well-suited to delineate the intricate MBEN microanatomy and capture cell-type-specific transcriptome profiles. We devise approaches to compare the sensitivity and specificity of different methods, along with their unique attributes, to guide method selection based on the research objective. Furthermore, we demonstrate how reimaging slides after the spatial transcriptomics analysis can significantly improve cell segmentation accuracy and integrate additional transcript and protein readouts, expanding the analytical possibilities and depth of insight.

**Conclusions:**

This study underscores important distinctions between spatial transcriptomics technologies and offers a framework for evaluating their performance. Our findings support informed decisions regarding methods and outline strategies to improve the resolution and scope of spatial transcriptomic analyses, ultimately advancing spatial transcriptomics applications in solid tumor research.

**Supplementary Information:**

The online version contains supplementary material available at 10.1186/s13059-025-03624-4.

## Background

Single-cell RNA sequencing of dissociated cells (scRNA-seq) or nuclei (snRNA-seq) has become a standard method in cancer research for dissecting deregulated transcriptional programs, as well as understanding cell types and cell fate trajectories [1]. However, in sc/snRNA-seq analyses, the spatial relationships between cells in their native tissue context are lost. Various emerging spatial transcriptomics (*ST*) approaches, which acquire molecular gene expression profiles of cells in situ, reveal the spatial relationships among individual cells [2–5]. *ST* technologies provide new insights into tumor heterogeneity and the interactions of tumor cells with their microenvironment [6, 7]. These methods can be broadly classified into sequencing-based (*sST*) and imaging-based (*iST*) approaches. The *sST* analysis employs a readout through sequencing after transcripts have been released from the sample and captured directly or via hybridized probes. This allows for an unbiased analysis of the whole transcriptome. The *iST* methods use multiplexed single-molecule RNA fluorescence in situ hybridization (smRNA-FISH) in a targeted manner, as defined by the probe panel, along with transcript identification through imaging. In *ST* experiments involving tumor samples, the different methods have their own specific strengths and weaknesses, and numerous questions remain regarding the optimal technical implementation of *ST* technologies and experimental design. On the one hand, the organism, tissue type, and sample processing methods (e.g., formalin-fixed, paraffin-embedded (FFPE), or fresh frozen tissue) will affect the results. On the other hand, there is currently no consensus on determining relevant parameters for quality control, including the following: (i) the *sensitivity* of the method, defined by the probability that a given transcript is detected; (ii) target *specificity*, as reflected by the false discovery rate (FDR); (iii) the specific genes and their total number that are adequately covered in the experiment; and (iv) the assignment of transcripts to individual cells.

The resolution of transcriptome analysis and cell type annotation depends on the experimental raw data, as well as their preprocessing and downstream analysis. For instance, a critical step in the workflow is segmenting cells for transcript assignment and cell type identification. Various dyes are available for staining nuclei, membranes, and whole cells, but results vary based on the organism, tissue type, and sample processing. Additionally, the microscopy system, such as wide-field versus confocal microscopes, along with the used objectives and detector sensitivity, influences image quality in terms of resolution and signal-to-noise ratio, thus affecting segmentation of nuclei and cells. Numerous computational methods, including Cellpose [8], Baysor [9], and Mesmer [10], have been developed for segmentation, with their results being highly dependent on the input data.

High-throughput *iST* commercial instruments use automated imaging and integrated microfluidics or pipetting robotics to complete the respective workflows. The following platforms were used in this study: (i) Molecular Cartography (MC) on the MC 1.0 instrument (Resolve Biosciences) [11], (ii) Multiplexed error-robust fluorescence in situ hybridization (MERFISH) [12] on the Merscope V1 (Vizgen) system [13], referred to as “Merscope” in the following, (iii) hybridization with barcoded padlock probes targeting RNA directly [14] as implemented on the Xenium Analyzer instrument (10x Genomics), which we refer to as “Xenium” [15]. Several reports compared the performance of these and other instruments for FFPE cancer tissue samples [16, 17] as well as mouse brain FFPE [18] and fresh frozen [19] tissue sections. However, a study using the same fresh frozen cancer samples across different *iST* platforms is lacking. Fresh frozen samples can be advantageous regarding RNA integrity and can facilitate unbiased single nuclei transcriptome and/or open chromatin profiling by scATAC-seq from the same sample.

Here, we applied a comparative *ST* analysis for a case study focusing on medulloblastoma tumors with extensive nodularity (MBEN) [20]. MBENs are a histopathologically defined subtype of medulloblastoma, which is among the most common embryonal central nervous system tumors in children [21, 22]. Due to mutations in the sonic hedgehog pathway, MBEN mimics the development of cerebellar granule neuronal precursors and thus features the complete developmental trajectory [20]. This is reflected in the MBEN tissue structure, which is characterized by a segregation into an internodular (proliferating cerebellar granular neuronal precursor-like malignant cells together with stromal, vascular, and immune cells) and nodular compartment (postmitotic, neuronally differentiated malignant cells). For our study, we used the same MBEN patient samples for MC, Merscope, and Xenium in comparison to RNAscope HiPlex (“RNAscope”) [23, 24] as a well-established reference for low-throughput *iST*. In addition, snRNA-seq and *sST* on the Visium platform (10x Genomics) were included as methods for an unbiased transcriptome analysis. Based on our experience with these six different methods, we identified informative QC parameters and metrics to assess sensitivity and specificity. Furthermore, we show how technological differences affect the results and provide guidance for the experimental design for the analysis of fresh frozen tumor samples by *iST*.

## Results

### ST of MBEN samples

**Fig. 1:**
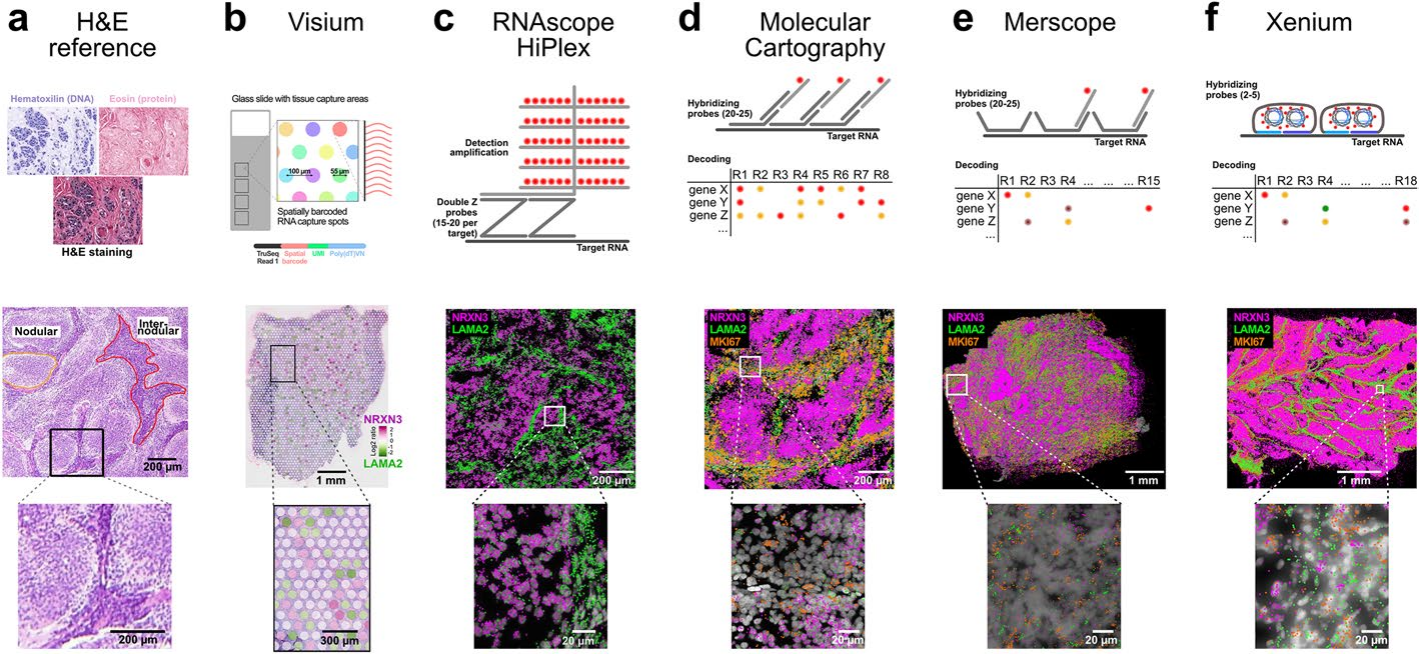
Overview of *ST* technologies compared in this study. Marker genes *NRXN3* (magenta, nodular compartment), *LAMA2* (green, internodular compartment), and MKI67 (orange, proliferating cells) are shown for sample MB295. **a** H&E reference staining [20]. **b** Visium. **c** RNAscope. **d** Molecular Cartography. **e** Merscope. **f** Xenium

The analysis of MBEN samples with their unique microanatomy and cellular compositions was performed on fresh frozen tissue sections from four distinct patients (Additional file 1: Table S1) using different *ST* methods (Fig. 1). These patients were previously studied with sequencing, microdissection, and spatial technologies [20].

Exemplary tissue images are presented for hematoxylin and eosin (H&E) staining (Fig. 1a) alongside the various *ST* technologies utilized (Fig. 1, Additional file 2: Fig. S1, Additional file 3: Table S2). These included Visium (Fig. 1b), RNAscope (10 gene panel, Fig. 1c), MC (100 gene panel, Fig. 1d), Merscope (138 gene panel, Fig. 1e), and Xenium (345 gene panel, Fig. 1f) (Supplementary Dataset 1). All *iST* panels encompassed the 10 genes from RNAscope, while MC, Merscope, and Xenium panels shared 96 genes. The MBEN tumor microanatomy is visible in the H&E staining, and its structure was highlighted by all *iST* methods at the transcript level, with the transcription of *NRXN3* and *LAMA2* as marker genes for the nodular and internodular compartments, respectively (Fig. 1). However, the Visium analysis did not offer sufficient spatial resolution to distinctly delineate the two tumor compartments, as indicated by the NRXN3/LAMA2 expression ratio (Fig. 1b). Additionally, we incorporated snRNA-seq data generated on the Chromium platform as a reference for a single-cell transcriptome analysis of solid tumor samples.

### *ST* image acquisition and reimaging of slides

For Visium and RNAscope experiments, image acquisition is decoupled from transcript detection and decoding. For Visium, H&E images were acquired on a slide scanner, and the RNAscope *iST* data was acquired by spinning disk confocal microscopy (SDCM). The commercial MC 1.0, Merscope V1, and Xenium instruments provide automated image acquisition on a built-in wide-field fluorescence microscope. The three systems differ in objectives, cameras, and preprocessing software (Table 1). All systems generally decode the transcript identities from fluorescent signals across several rounds of staining, imaging, and destaining and yield transcript coordinates and a matching tissue map as a DAPI image.

**Table 1:** Feature overview of automated *iST* platforms

	**MC**	**Merscope**	**Xenium**
Detected features per cell^a^	21 ± 2	23 ± 4	25 ± 1
Detected transcripts per cell^a^	74 ± 11	62 ± 14	71 ± 13
Correlation with RNAscope^b^	*r* = 0.74	*r* = 0.65	*r* = 0.82
Features with cumulative transcript counts in range of background signal^a,c^	29 ± 8	43 ± 2	18 ± 2
Average FDR (%)	0.35 ± 0.2	5.23 ± 0.9	0.47 ± 0.1
Probes with low specificity^d^	12 ± 3	17 ± 3	7 ± 3
Reimaging	Yes^e^	No	Yes
Run time instrument (days)	4	1–2	2
Hands-on time to prepare slides(days)	1.5	5–7	1.5
FWHM (0.31 µm beads) (nm)^f^	352 ± 50	480 ± 85	474 ± 55
Magnification	50x	60x	Not provided
Numerical aperture (NA)	1.2	1.4	Not provided
Pixel size (nm)	138	108	212
*z*-stacks distance (µm)	0.36	0.7	0.75
*z*-stacks number	32	7	48
*z*-stacks number DAPI	32	7	12 (every 3 µm)
Imaging rounds	8	18	15
Colors	2	3	4
Optimization parameters^g^	Quenching	Tissue clearing and bleaching	NA
Minimal distance between transcripts (in one *z*-plane)	138 nm (= 1 pixel)	3.4 nm	2.6 nm

^a^Data are averages of median values and their standard errors and refer to the shared 96 gene panel

^b^Based on 10 gene panel shared with RNAscope

^c^Different background probes were used for the different technologies

^d^Probes that displayed a signal intensity in the range of the background value had low spatial autocorrelation and a higher minimal distance to its nearest neighbor as described in the text

^e^Slide is glued to chamber

^f^Reported as median ± median absolute deviation (see the “Methods” section for details)

^g^Experimental steps that might require sample-to-sample optimization

To compare the resolution of the *iST* platforms, we imaged 0.31 µm multicolor fluorescent particles across all systems. We analyzed the full width at half maximum (FWHM) of the beads (Additional file 4: Fig. S2). For comparison, we included SDCM images obtained with 40× and 60× oil immersion objectives under the same conditions used for RNAscope and reimaging, respectively. Overall, the observed FWHM was very similar for Xenium (474 ± 55 nm) and Merscope (480 ± 85 nm). For MC, the FWHM was only 352 ± 50 nm, which could indicate a deconvolution step in the onboard image processing pipeline before image stitching. The optical resolution of the automated microscopy systems limits the separation of crowded (highly expressed) transcripts of the same gene and the total number of transcripts that can be detected. In contrast, transcripts of different genes can be separated even when localizing to the same pixel. This is achieved through an intelligent combinatorial barcoding codebook design, along with a sufficiently large number of imaging rounds and colors, as well as the inclusion of the *z* coordinates for decoding. This holds true for Xenium and Merscope, where we found a minimal distance between any two transcripts that was below one pixel. For MC, the minimal distance between any two transcripts was larger than one pixel (Table 1).

The *iST* systems used in this study implement different smRNA-FISH protocols. The most prominent difference is the presence (Xenium, RNAscope) or absence (MC, Merscope) of secondary signal amplification and clearing of the tissue section (Merscope only). To estimate the signal-to-noise and signal-to-background ratios, we also analyzed actual transcript spots in single-tile *z* stacks (Additional file 5: Fig. S3a). Unfortunately, these are not provided for Xenium (even upon request to 10x Genomics) and are, therefore, missing from this assessment. As we could not match the provided raw data (single tile stacks) confidently to the actual transcript coordinates, spots on the images were called using RS-FISH [25]. Then, we analyzed line profiles (*x*-direction) spanning 10 pixels on either side of the spot center. The background levels were more homogeneous for Merscope as expected due to the clearing step. However, the signal-to-noise ratio was slightly higher for MC and even higher, but also more variable for RNAscope (Additional file 5: Fig. S3b). Last, the signal-to-background ratio was higher and more variable for RNAscope as expected for a method relying on secondary signal amplification. Overall, we conclude that the experimental procedures for all analyzed methods result in spots that can be readily separated from background. We anticipate that the major challenge lies rather in the reidentification of the same spots across all imaging rounds required for decoding the combinatorial barcodes.

After the *ST* run, conducting a reimaging step may be beneficial to acquire higher resolution images (e.g., improved DAPI images for cell segmentation, see below) or to include additional imaging modalities such as H&E, membrane, or immunostaining. For MC and Xenium, this process is straightforward since tissue integrity is preserved during the runs and standard slide formats are utilized (Additional file 2: Fig. S1).

**Fig. 2:**
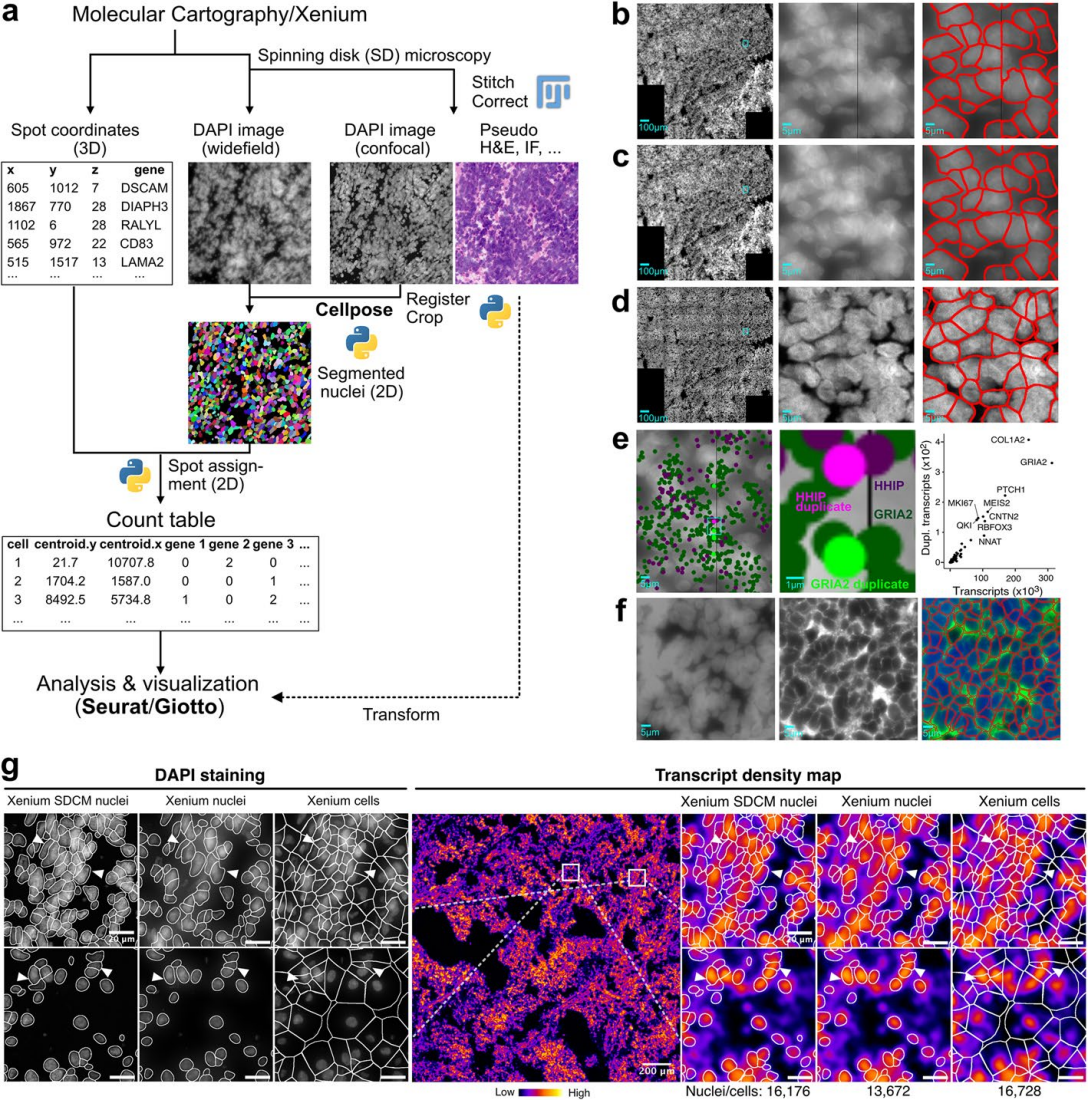
Reimaging and segmentation. **a** Reimaging workflow for MC and Xenium slides. **b** Widefield overview DAPI image, zoomed-in and segmented image for MC. **c** Same as panel **b** after applying the MindaGap software to fill the non-overlapping line between images. **d** Same region as panels **b** and **c** after reimaging with the spinning disk microscope. **e** The gap between images introduces artifacts in stitching and registration, leading to the artificial generation of duplications for 0.15% of the transcripts. **f** Segmentation for Merscope with membrane staining. Left: DAPI-stained wide-field image; middle: membrane staining; right: segmentation based on DAPI signal and membrane staining. **g** DAPI images (zoomed in on the indicated regions) of the Xenium slide and segmentation based on SDCM and widefield images for tumor tissue MB266. The region overview is represented as a transcript density map. The fraction of transcripts assigned to segmented nuclei or cells was 68% for Xenium SDCM nuclei (Cellpose-based segmentation of Xenium slide reimaged by SDCM), 59% for Xenium nuclei (Cellpose-based segmentation on the original Xenium image), and 95% for Xenium cells (original segmentation provided by the Xenium workflow). The cell expansion used for the latter segmentation covers nearly all transcripts. However, this is associated with artifacts, as indicated for the cells marked with arrowheads. For quantification, see Additional file 6: Fig. S4

We have developed a workflow for MC and Xenium that involves reimaging the slides using SDCM, with the resulting images registered to the original wide-field DAPI images obtained from the automated systems (Fig. 2a). Reimaging of Merscope slides was not tested because the sample clearing step prior to the run removes all tissue components except RNA and DNA. To overlay images of the tissue obtained from a different microscope, images (maximum intensity projections) were first stitched together and then registered to the DAPI images from the *iST* analysis of the MC or Xenium system. This approach allows for the integration of the cells’ transcriptome profile with additional readouts.

Although stitching and registration are generally seen as standard image processing tasks, they can be challenging in practice due to several factors. First, multiple rounds of transcript detection (MC: 8, Merscope: 18, Xenium: 15) involving washing, bleaching, and recoloring can compromise tissue integrity, making reimaging to enhance the DAPI signal ineffective. While we have not observed this in our MBEN samples, we do not rule it out for other tissue types or additional staining procedures (e.g., for proteins or DNA). Second, registration becomes difficult when the tissue is not firmly attached to the slide, causing deformation during the imaging processes, or when stitching is inaccurate on either the registration source or template. We have rarely noticed deformation, and when it does occur, it is mostly limited to single cells rather than large tissue areas. Stitching artifacts were an issue for MC (see below), but these could be resolved by using elastic transformations instead of affine ones. Lastly, evaluating the quality of the registration results is difficult because the intensity distributions across the tissue and even within single cells vary significantly between confocal and widefield images, rendering correlation metrics unsuitable. Practically, we performed a pairwise overlay of the DAPI images from different modalities and visually inspected the results.

### Image processing and cell segmentation

To assign transcripts to individual cells after segmentation, several image processing steps were performed. Unless noted otherwise, we used segmentation based on DAPI staining (MC), DAPI and membrane staining (Merscope), and DAPI staining with cell expansion (Xenium) as the default workflows for the various systems. We find that the quality of DAPI images is critical and strongly relies on experimental factors (tissue quality and staining) as well as technical aspects, namely image acquisition parameters (excitation intensity, exposure time, dynamic range of the detector), image resolution and out-of-focus signal for the wide-field microscopes used in these systems. Therefore, it is essential to optimize the DAPI signal-to-noise ratio and to avoid too low a signal as well as overexposure.

The DAPI image quality obtained with the default settings of automated *iST* systems can be insufficient for resolving single cells in the highly cell-dense MBEN tissue sections. To evaluate the impact of this issue for the subsequent preprocessing and segmentation methods, we also acquired SDCM images for MC and Xenium (referred to as “MC SDCM” and “Xenium SDCM”). In cell-dense regions, analysis of the original wide-field images can yield ambiguous results. This is demonstrated for the MC workflow in Fig. 2b–d. The analysis also highlighted stitching artifacts caused by non-overlapping images, resulting in black strips that bisect cells spanning across imaging tiles (Fig. 2b). On the image analysis side, Gaussian blurring can mitigate this issue (Fig. 2c). Still, the inaccurate stitch borders may lead to duplicate transcripts. To further explore this concern, the confocal images were registered, and the transcript duplicates were eliminated (Fig. 2d). These duplicates were identified at a low but still detectable frequency of 0.15% across all transcripts (Fig. 2e). It is important to note that inaccurate stitch borders in the registration template (i.e., the original DAPI image) hinder the use of robust affine registration models in most cases, as they result in systematic shifts across the large dimensions of the imaged areas. Instead, we employed elastic registration with bUnwarpJ in Fiji [26], which locally adjusts the images at inaccurate stitch borders, yielding excellent results despite being less robust and more contingent on initial registration parameters. This type of stitching error was not observed with Merscope and Xenium systems. Lastly, the option available for Merscope to incorporate membrane staining into the standard workflow can enhance cell segmentation in wide-field areas, as illustrated in Fig. 2f. The same objective can be addressed for Xenium with the newly introduced multi-tissue stain mix [27]. It combines nuclear (DAPI), membrane (ATP1A1, E-Cadherin, CD45), cytoplasmic (18S rRNA), and cytoskeletal (αSMA, Vimentin) stains to facilitate cell segmentation.

In general, segmentation with Cellpose [8] using the DAPI signal yielded good results, and the SDCM images showed a 15–30% higher count of segmented nuclei. This is demonstrated for sample MB266 in Additional file 6: Fig. S4a–d. Approximately 71% (MC) and 68% (Xenium) of the total detected transcripts were found within the segmented nuclei. In contrast, nuclei segmentation on the corresponding widefield image resulted in roughly 10% fewer assigned transcripts to nuclei (MC, 58%; Xenium, 59%). This discrepancy can be partly attributed to the lower total number of segmented nuclei in the widefield images (~ 28% for MC and ~ 15% for Xenium in the case of MB266). However, it is important to note that not only does the number of segmented cells or nuclei matter, but also their size and shape. More transcripts were detected in larger cells. Furthermore, the area covered by segmented cells/nuclei also needs to be considered. Simply extending segmented nuclei to include cytoplasmic transcripts led to some incorrectly assigned transcripts, resulting in a mixed transcriptome from different cells (Fig. 2g). Notably, the improved segmentation result from SDCM DAPI reimaging does not stem from higher resolution per se (Additional file 4: Fig. S2), but rather from the suppression of out-of-focus light in confocal microscopy, which yields more contrasted and detailed nuclei (Additional file 6: Fig. S4e–f).

### Sensitivity of *ST* methods

**Fig. 3:**
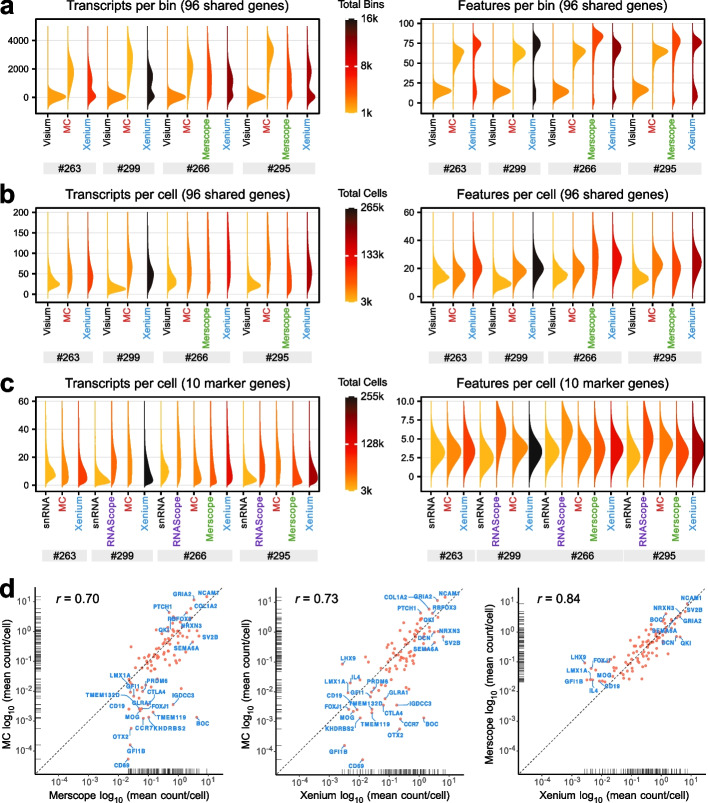
Sensitivity of *ST* methods. **a** Density ridge plots showing transcript and feature counts per spatial bin (48.74 µm side length square), equivalent to the area of one Visium spot. **b** Density ridge plots of transcript and feature counts per cell after segmentation for 96 shared genes. **c** Same as panel **b**, but for the 10 shared genes included in the RNAscope panel. **d** Correlations of transcript counts between different *iST* methods, with the dashed line indicating the equivalent number of transcripts detected for the two methods compared. Correlations of automated *iST* with snRNA-seq data are provided in Additional file 7: Fig. S5b–d

The sensitivity of *ST* methods can be defined as the fraction of transcripts detected. To assess this parameter, we analyzed the distribution of the total number of transcripts detected (“transcripts”) as well as the number of genes (“features”) for the shared 96 gene panel common to all *ST* methods except RNAscope. To eliminate the confounding effect of segmentation, we conducted a spatial binning analysis as a segmentation-free approach. The number and type of transcripts were determined within spatial bins (48.74 × 48.74 µm) that correspond to the area of a circular Visium spot, which is approximately 2375 µm^2^ in size. The *iST* techniques clearly outperformed the Visium *sST* method regarding the number of transcripts and features in this comparison (Fig. 3a). While increasing the sequencing depth might improve the Visium results, we still expect that Visium’s sensitivity will remain considerably lower than that of the *iST* techniques. Among the latter group, MC yielded the highest number of transcripts per bin, while the number of features was similar across all automated *iST* methods. Interestingly, the spatial binning analysis yielded a bimodal distribution for Xenium, indicating the presence of tissue regions with decreased transcript coverage. The bins within the lower transcript distribution were enriched in the outer regions of the tissue in line with the observation that tissue borders often suffer more damage during freezing and cutting processes (Additional file 7: Fig. S5a).

Next, the number of transcripts or features was calculated per cell for the 96 gene set (Fig. 3b). This comparison revealed only minor sensitivity differences among the automated *iST* instruments. The well-established RNAscope method consistently produced high numbers of transcripts/features per cell for the 10 shared genes (Fig. 3c). This result confirms its use as an smRNA-FISH reference in the field. We then conducted a pairwise comparison of the detected mean transcripts per cell across the different *iST* methods for the shared gene set (Fig. 3d). Some genes with lower expression levels, such as *BOC*, appeared to be better detected on a per-cell basis by Merscope and Xenium compared to MC. This might also stem from variations in the segmentation protocol, as the extension of the cells in the Xenium analysis results in nearly complete coverage of the tissue area.

The same analysis was applied in reference to snRNA-seq, which has a reported detection efficiency of 14–15% for the Chromium 3′-RNA v2 chemistry (10x Genomics) used in our experiments (Additional file 7: Fig. S5b–d). The resulting correlation coefficients of *iST* methods with snRNA-seq were between 0.53 and 0.63, which is somewhat lower than the coefficients between the *iST* methods themselves, ranging from 0.7 to 0.84 (Fig. 3d). On average, the number of a given transcript per nucleus or cell was 2.3- to 2.5-fold higher for the *iST* methods than for snRNA-seq (Fig. 3b, Additional file 7: S5b–d). This suggests that the detection efficiency of the *iST* methods is approximately 33–37%.

Overall, the automated *iST* methods produced very similar results, with MC showing a slightly lower count of less abundant transcripts per cell when using different default segmentation methods. When assessing the number of transcripts and molecules per cell, all *iST* techniques demonstrated higher sensitivity than snRNA-seq performed with the Chromium v2 chemistry.

### Specificity of automated *iST* methods using RNAscope as a reference

**Fig. 4:**
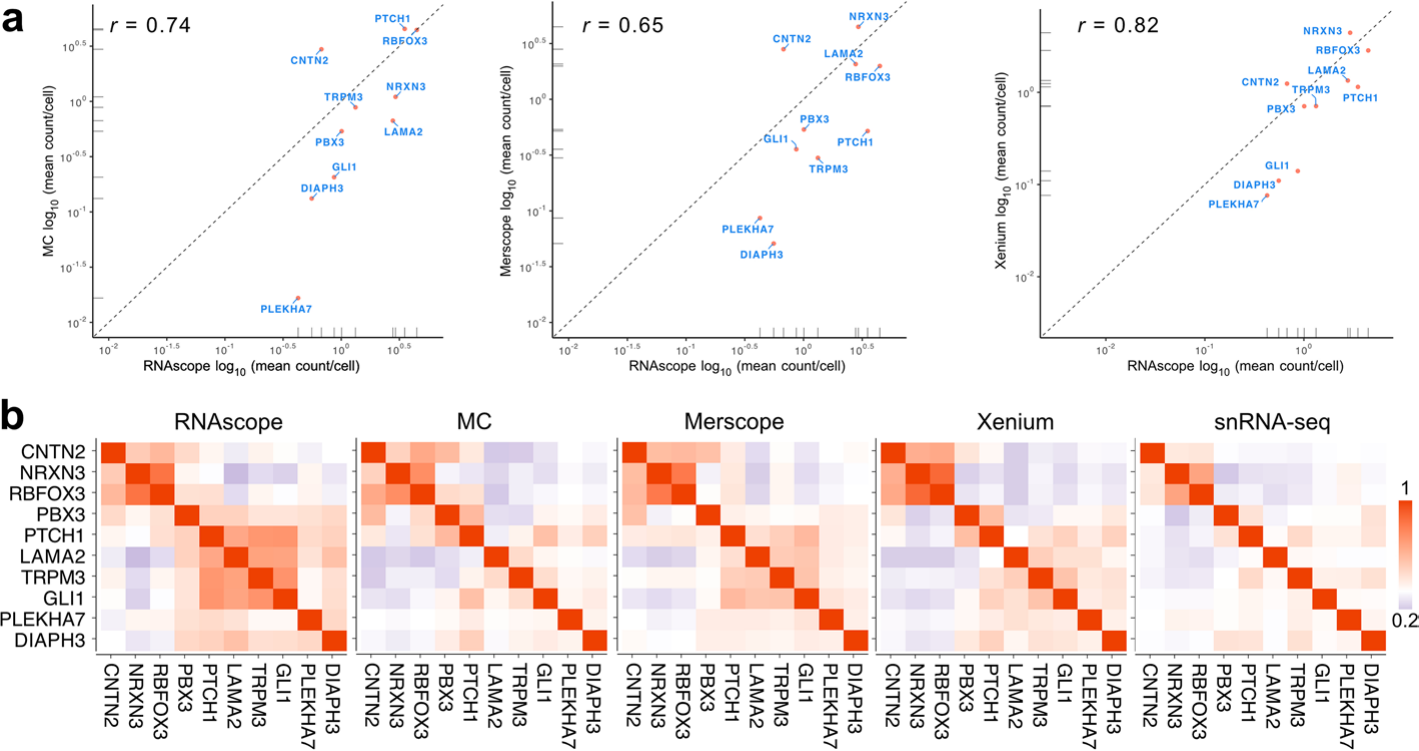
Comparison with RNAscope. **a** Correlation of gene expression across different automated *iST* methods with RNAscope for the 10 shared genes. The dashed line represents the same number of transcripts detected by the two methods being compared and indicates that 7 out of 10 (MC) and 8 out of 10 genes (Merscope, Xenium) exhibited a higher number of transcripts per cell/nucleus, while *CNTN2* was identified more effectively with all automated *iST* methods. **b** Analysis of marker gene co-expression from the pairwise Pearson correlation coefficient

To assess *ST* specificity, we used the 10 genes mapped in the RNAscope data as a reference. We calculated the correlations of the mean number of transcripts per cell for these 10 genes. The highest correlation was found between RNAscope and Xenium (Fig. 4a). Next, we computed pairwise correlation coefficients for transcripts within a cell for each of the different methods (Fig. 4b).

This correlation analysis indicated that the RNAscope data accurately reflected the MBEN tissue microanatomy described in ref. [20]. Marker genes of the nodular compartment (*RBFOX3*, *NRXN3*) and those of the internodular compartment (*GLI1*, *TRPM3*, *LAMA2*, and *PTCH1*) showed high positive correlations within their respective groups but were neither correlated nor anti-correlated between the two groups. The Merscope data was most similar to the pattern of (anti-)correlations between gene pairs observed in the RNAscope data (coefficient of determination *R*^2^ = 0.72), while this pattern was somewhat less apparent for the other methods (MC, *R*^2^ = 0.45; Xenium, *R*^2^ = 0.58) (Additional file 8: Table S3). This type of assessment relies on prior knowledge about the spatial expression patterns of a given tissue and can be implemented after cell segmentation as a quality assessment for specific marker genes that exhibit distinct spatial relations, as demonstrated here for MBEN.

### Specificity of *iST* methods inferred from background probes

**Fig. 5:**
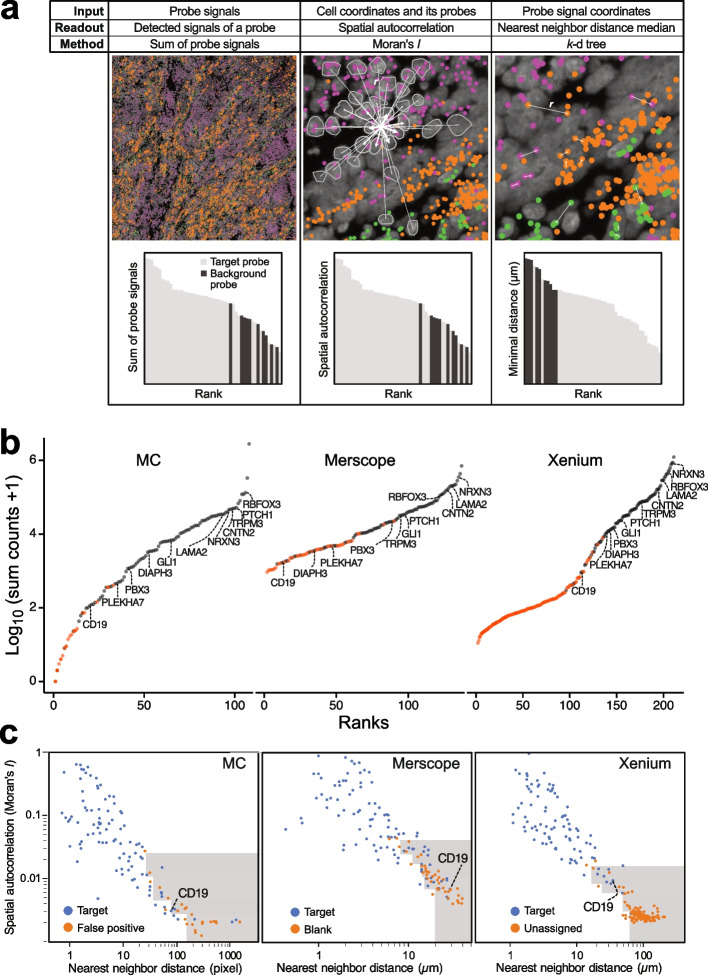
Specificity analysis comparing target and background probes. Data are shown for MB266. **a** Approach for the specificity analysis across different length scales. Coordinates for three different transcripts are represented in purple (*NRXN3*), green (*LAMA2*), and orange (*MKI67*). *Left*: whole tissue analysis with target and background probes ranked by summing all signals detected for each probe. *Middle*: spatial autocorrelation of probe signal calculated using Moran’s *I*. This value increases if a given cell’s signal (indicated by outgoing distance vectors) is similar to the average signal of neighboring cells at distance *r*, as shown by the connecting vectors, weighted by 1/*r* between two cells (vector thickness indicates greater weights). *Right*: minimal distance to the nearest probe signal of the same type. This parameter helps to identify clusters at subcellular resolutions arising from rare and isolated cell types. Exemplary pairs of transcripts are marked with white arrows. **b** Ranked target and background sum counts for all targets and the corresponding technology-specific background probes (see Additional file 9: Table S4, Additional file 10: Fig. S6). **c** Analysis of the spatial distribution of target and background probes. Moran’s *I* (scaled from 0 to 1) was plotted against the median nearest neighbor distance. Higher values of Moran’s *I* and lower nearest neighbor distances indicate a non-random distribution. The gray area highlights low confidence probes based on the 0.05 percentile of the nearest neighbor distance within a given range of Moran’s *I*

Next, we assessed specificity by relating the signal from fluorescently labeled control probes, referred to as background probes here, which lack a complementary sequence in the sample across different length scales (Fig. 5). It is important to note that the manufacturers supplied the background probes, and information regarding their sequences is lacking. The three *iST* methods also utilize different controls (Additional file 9: Table S4). MC and Merscope rely on the binding of numerous probes to achieve a sufficient signal. Consequently, false positive signals typically arise from the read-out probes rather than the primary probes, as a single, incorrectly bound primary probe yields fluorescence signals below the detection limit and will therefore be “invisible”. In the case of Xenium, due to the amplification of the signal from a single padlock probe, both off-target binding of primary and secondary probes must be considered. Accordingly, unspecific primary probes are also included in the kit.

By comparing the sum of all signals from a given probe across the entire tissue, we identified 29 ± 8 (MC), 43 ± 2 (Merscope), and 18 ± 2 (Xenium) probes for which the signal range fell within that of the background probes (Fig. 5b). Among these, the genes *GFI1*, *LMX1 A*, *IL4*, *FOXJ1*, *CD19*, *TMEM119*, *MOG*, *CD69*, and *GFI1B* exhibited consistently low expression values across all three automated *iST* technologies. This could indicate true negative signals or RNAs that are difficult to target, e.g., due to stable secondary structures. Based on the evaluations of target and background probes, we calculated global, segmentation-free FDR values of 0.41 ± 0.2% (MC), 5.23 ± 0.9% (Merscope), and 0.47 ± 0.1% (Xenium). According to these average global FDR estimates, the specificity is quite similar for MC and Xenium, with Merscope showing a higher FDR value.

The average FDR value does not account for specific signals that are simply low in abundance. Therefore, we evaluated the spatial distribution of the target probes (Fig. 5a). We computed their spatial autocorrelation using Moran’s *I* [28, 29] and assessed the minimal distance between probe signals targeting the same RNA. The analysis of Moran’s *I* was based on the premise that a false positive signal due to technical issues would be randomly distributed (*I* = 0). In contrast, a lowly abundant true positive signal (for example, a lowly expressed marker for a niche cell type) would exhibit some enrichment (*I* > 0) and/or display clustering at the (sub)cellular level within isolated rare cell types that would result in a low minimal distance. By combining spatial autocorrelation signals with nearest neighbor distances, specific cut-offs can be established to identify targets that exhibit a lowly abundant signal that is not randomly distributed within tissue space (Fig. 5c, Additional file 10: Fig. S6a).

The spatial autocorrelation analysis was performed at the resolution of individual cells and their neighboring cells, while the distance to a transcript’s next nearest neighbor also encompasses subcellular distances. This distance tends to be small for transcripts primarily located in isolated rare cell types scattered throughout the tissue sections. We applied a 0.95 percentile cut-off for a given Moran’s *I* range, with four distinct ranges for each technique based on Supplementary Dataset 2. The number of confident transcripts increased for all techniques compared to the expression level analysis. On average, 7 ± 3 (Xenium), 12 ± 3 (MC), and 17 ± 3 (Merscope) transcripts did not meet the threshold (as demonstrated in Fig. 5c). This indicates a slightly noisier signal for the latter method, which aligns with its higher average FDR value.

We also examined the spatial distribution of target and background probes in relation to the segmented area by measuring the ratio of probe signals inside and outside nuclei. This analysis yielded similar results for both target and background probes across technologies and tissue samples, even when using nuclear segmentation with no expansion (Additional file 10: Fig. S6b,c). Thus, the majority of the signals from both target and background probes were located in the nucleus. Finally, we explored the distribution of detected molecules across the recorded *z*-levels for each technology. While MC and Xenium yield images for a wide range of *z*-values from below to above the actual sample, detected molecule numbers were high for all seven *z* planes for Merscope, and the distribution appeared to be cut off (Additional file 10: Fig. S6 d). This suggests that a significant number of molecules above and below the seven imaged planes in Merscope remain undetected.

### Detection of cell types across platforms

**Fig. 6:**
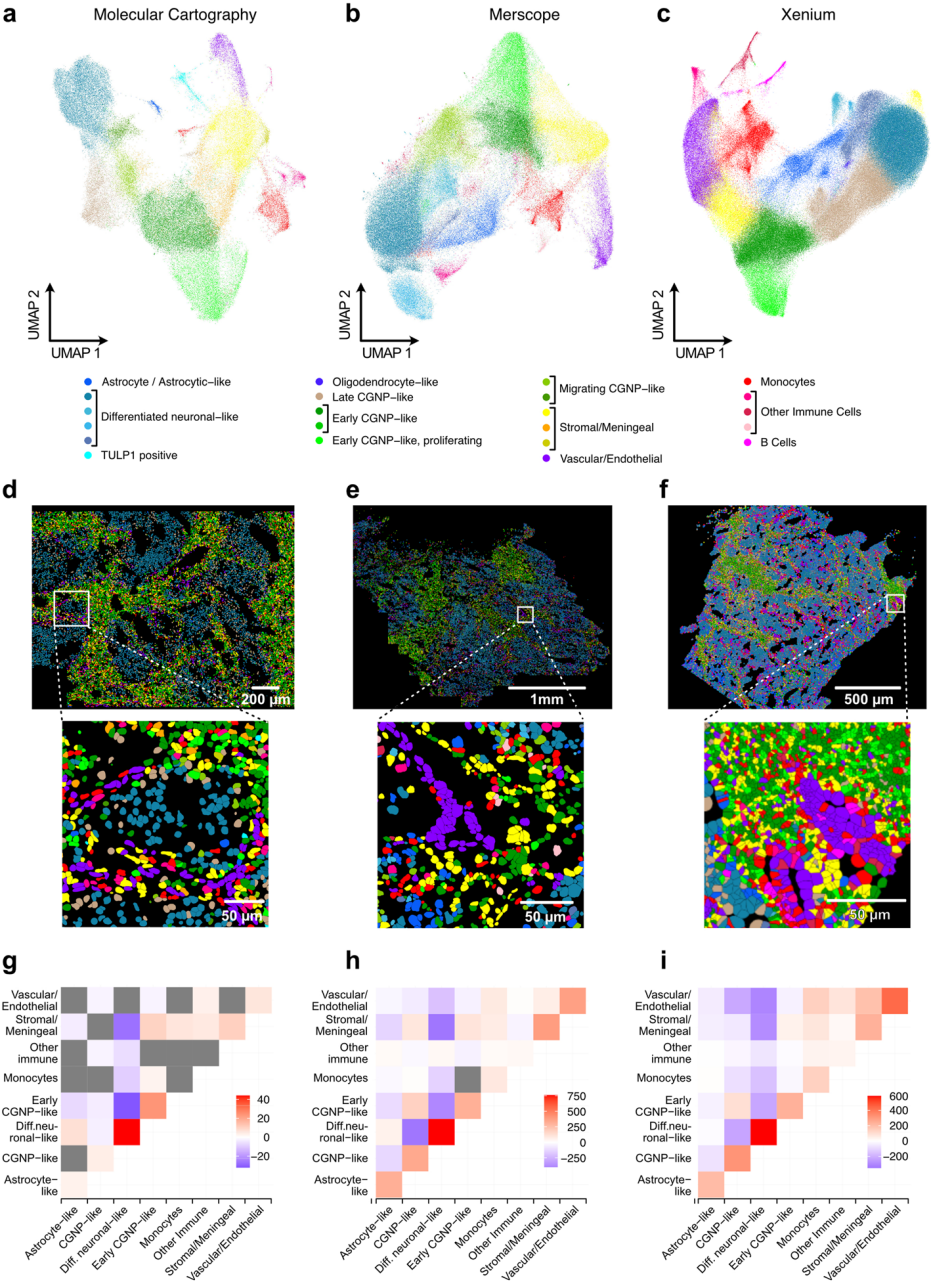
Clustering and cell type annotation for *iST* methods. Clustering was based on the shared set of 96 genes in samples MB266, MB295, and MB299. The joint cell type annotation relied on the expression signatures of the different clusters described in Additional file 11: Fig. S7. **a** Clustering and UMAP visualization for MC (*n* = 106,403 cells). **b** Same as panel **a,** but for Merscope (*n* = 139,916 cells, MB266 and MB295 only). **c** Same as panel **a**, but for Xenium (*n* = 569,152 cells). **d** Images with cells colored according to their cluster. **e** Same as panel **d**, but for Merscope. **f** Same as panel **d**, but for Xenium. **g** Heatmap showing the *z*-scores of the enrichment or depletion of pairs of neighboring cell types compared to random distribution for MC with non-significant interactions shown in gray. **h** Same as panel **g**, but for Merscope. **i** Same as panel **g**, but for Xenium

To compare cell type identification across different technologies, we followed standard clustering workflows, assigned cell types based on the expression signatures identified in our previous MBEN study [20], and visualized the data using UMAPs (Fig. 6a–c, Additional file 11: Fig. S7). The overall cell type annotations were very similar for the *iST* methods, and the same major cell types were found across all platforms. However, inspection of the cluster heatmaps revealed differences in the detection efficiency of single transcripts that affect cell type assignment (Additional file 11: Fig. S7 d–f, Supplementary Dataset 3). For example, a *TULP1-*positive cell type (MC), a differentiated neuronal-like cell type marked by *KHDRBS2 (Merscope),* and *CD19+* (Xenium) were specific for the three different platforms. Thus, cell type annotations differ between technologies mainly due to lowly abundant transcripts detected better on one platform than on another. For example, as illustrated in Fig. 5b and c, the specificity of CD19 detection was similar to that of the background probe, which demonstrates the need to consider this technical aspect for cell type assignment.

The “differentiated neuronal-like” annotation revealed subclusters for both Merscope and Xenium, while cell subtypes were identified for the stromal compartment by MC. Determining whether these subclusters represent distinct cell types or states requires further investigation. The spatial distribution of cell types, along with the corresponding coloring, is shown for exemplary images and regions in Fig. 6d–f.

Next, we assessed the ability of the three automated *iST* platforms to capture spatial relationships between cell types by conducting a neighborhood analysis (Fig. 6g–i). All three platforms enabled the identification of three distinct neighborhoods: the nodular compartment containing astrocytic-like and differentiated neuronal-like cells, the internodular compartment characterized by cerebellar granule neuronal precursor (CGNP)-like cells, and a stromal neighborhood enriched with stromal, vascular, and immune cells. These patterns appeared more distinct in Xenium and Merscope, likely due to the larger image area and cell number compared to MC. Finally, we evaluated the impact of using nuclear segmentation after reimaging with SDCM (Fig. 2g) on this analysis for two Xenium slides (MB266 and MB295, Additional file 12: Fig. S8).

While the major cell types (Fig. 6g–i) and their expression profiles were not drastically changed between the two different segmentations, some distinct differences were observed (Additional file 11: Fig. S7f, Additional file 12: Fig. S8b): (i) B cells and oligodendrocyte-like cells were undetected in the re-analysis. This is likely because they are relatively rare cell types (0.81% and 0.27%, respectively) and the reanalysis was conducted only on smaller parts of the tissue sections. (ii) Late CGNP-like and stromal/meningeal clusters display distinct subclustering in the re-analysis. As mentioned above, the relevance of these distinctions needs to be analyzed further. (iii) A population of migrating CGNP-like cells was identified by SDCM imaging and nuclear segmentation that could not be detected in the standard Xenium data. These migrating CGNP-like cells were concentrated in dense tissue areas, suggesting that improved imaging and segmentation lead to differences in transcript assignment that are particularly relevant for identifying cell types in the cell-dense regions of the tissue (Additional file 12: Fig. S8c–e).

### Implementation of additional readouts after *iST* analysis

While the *ST* analysis offers a wealth of information on molecular cell profiles in their spatial tissue context, corresponding studies typically require integration with other readouts. Merscope enables the inclusion of protein co-detection through up to five oligo-conjugated antibodies. Similar methods have been announced for the MC and Xenium platforms. A frequently used alternative is to prepare consecutive tissue sections for *ST* and other readouts. However, in many cases, the cell-by-cell assignment of these consecutive sections can be cumbersome and effective only in certain areas. Other approaches involve conducting additional readouts on the same tissue by either reimaging and subsequent image registration (MC and Xenium) or incorporating extra custom readouts directly into the *ST* run (Merscope). This is described here for three examples.

**Fig. 7:**
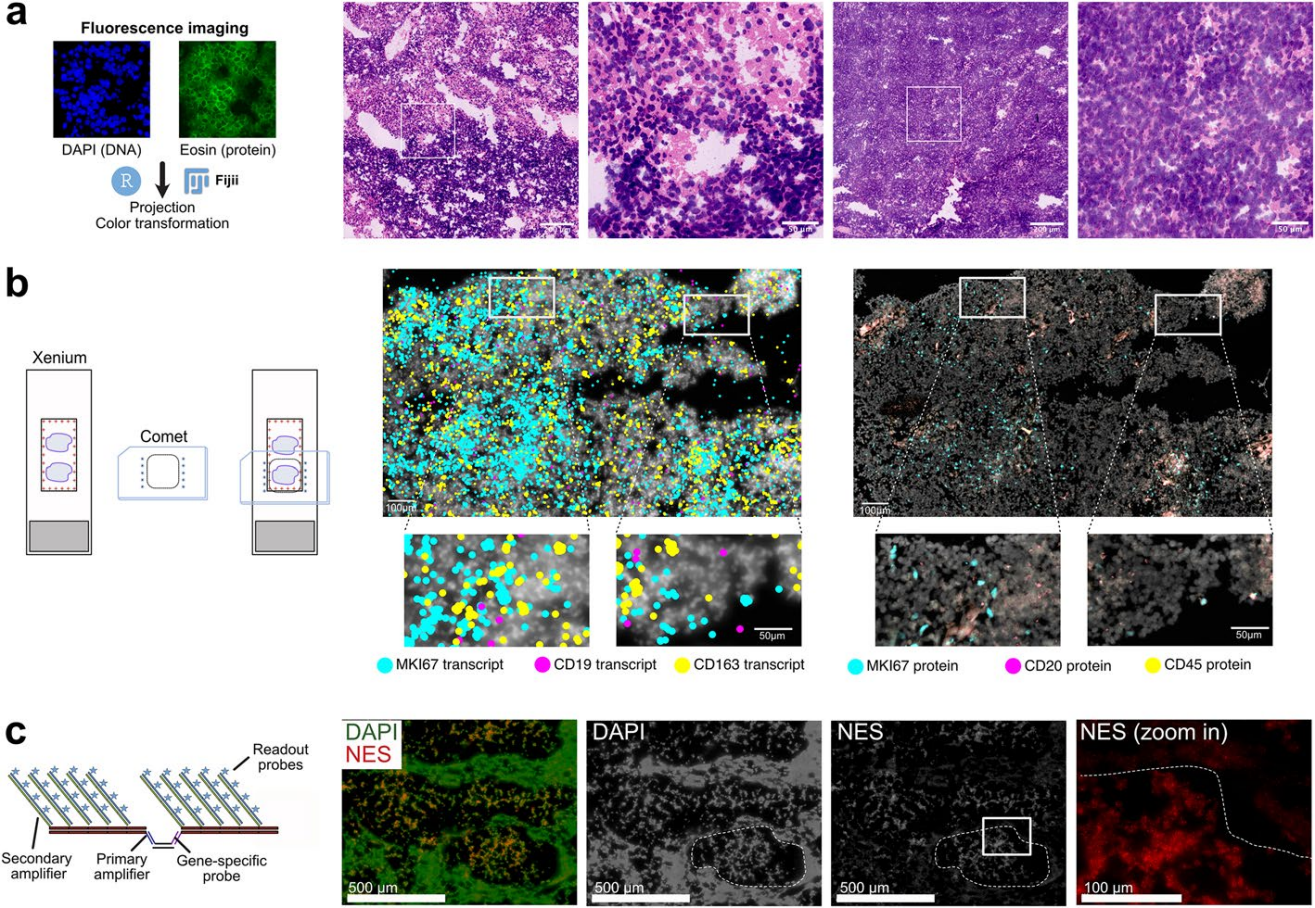
Additional readouts after *iST* analysis. **a** Virtual H&E staining of MBEN tissue after MC run. **b** Immunostaining on the Comet system after the Xenium run. Transcript signal is given as a single dot while the protein image reflects the original fluorescent signal. **c** Amplified readout of nestin (*NES*) as an exemplary custom RNA via the auxiliary channel on the Merscope

The first step involves virtual H&E staining of tissue following the MC run (Fig. 7a). Conventional H&E staining after the *iST* run is compatible with both MC and Xenium; however, it will hinder the subsequent acquisition of additional fluorescence signals, as the broad absorbance spectrum of hematoxylin interferes with nearly all fluorophores in the visible spectrum. This limitation can be bypassed by imaging DNA through DAPI staining (λ_ex_ = 405 nm, λ_em_ = 445 ± 23 nm) and eosin (λ_ex_ = 488 nm, λ_em_ = 521 ± 19 nm), followed by transforming these signals into a virtual H&E staining (Fig. 7a) [30]. Factors such as cell type-specific shades of pink produced by eosin in brightfield images or variations in cell autofluorescence cannot be fully addressed with the method used here. Nevertheless, it provides a valuable approximation whose applicability must be evaluated for each specific use case.

In Fig. 7b, we demonstrate that the Xenium slide can also be utilized for subsequent multiplexed immunostaining (up to 40 targets) on the Comet platform from Lunaphore (Additional file 13: Table S5). For our test case, we validated the presence of *CD19+* B cells, which were detected on the Xenium but not on the MC and Merscope systems. By employing immune (CD45) and B-cell (CD20) specific antibodies, along with Ki67 to stain cycling cells on the Comet system after a Xenium run, the immunostaining confirms the presence of CD19^+^/CD20^+^ B cells at both the transcript and protein levels.

The third example is the detection of a custom gene using the Merscope with signal amplification [31] (Fig. 7c). Overhangs on the primary probe are hybridized by the primary amplification probe, which then serves as a binding site for the secondary amplifier (Additional file 14: Table S6). The secondary amplifier can be detected using auxiliary probes in the Merscope chemistry. Nestin (*NES*), as an exemplary custom RNA target, was identified via an auxiliary probe on the Merscope and showed enrichment in the nodular structure (dashed outline). Signal amplification allowed for the detection of *NES* using only two primary probes instead of 30 in the original workflow. This workflow could potentially be used to detect short transcripts or gene fusions. Additionally, these auxiliary probes may also facilitate protein co-detection through oligo-conjugated antibodies, which we did not test in our experiments.

In summary, all three automated *iST* platforms allow for the integration of protein readouts, although MC and Xenium offer somewhat greater flexibility since the overall tissue structure remains intact during the experiment. However, it is important to note that the *iST* platforms use different tissue fixation chemistries, which may require antibody testing before conducting the experiments.

### Selecting a platform for a specific application

To guide selecting a method for a specific application, we compared key features of automated *iST* platforms (Table 1). Based on our experience, each platform presents unique advantages tailored to various research goals. MC is particularly effective for analyzing a limited number of samples, offering a versatile format for validation experiments, with its standard reagent kit supporting 8 tissue sections per slide and a panel of 100 custom genes. Merscope stands out with larger tailored panels (up to 960 genes), while Xenium is designed for large-scale datasets, facilitating the analysis of many samples or large tissue areas.

From a practical implementation perspective, MC and Xenium showcase user-friendly protocols with straightforward washing and incubation steps. In contrast, Merscope requires more extensive hands-on time and optimization for essential steps, including tissue clearance, quenching, and gel formation, which carry the risk of sample loss if detachment occurs. While the microscope and liquid handling systems across all platforms generally performed reliably, we noted specific technical challenges: MC occasionally encountered issues with sample transport and liquid handling due to its modular robot arm setup. In this system, readout probes are removed after each imaging round, allowing the entire workflow to be restarted in case of a power outage or system malfunction. This option is not available for Merscope, as the fluorophores become irreversibly bleached during imaging and are not removed. Merscope required meticulous objective alignment and readjustment, and Xenium sometimes experienced dispensing or data processing issues. Overall, Merscope exhibited the fewest aborted runs during our trials, probably due to the microfluidic-based liquid management. However, we emphasize that these technologies are rapidly evolving, and our observations reflect only current implementations. Based on our comparative analysis, researchers should prioritize the following key factors when selecting a platform:

1. *Reimaging capabilities*: Reimaging can significantly improve cell segmentation accuracy, which is particularly critical for 3D applications. Xenium’s limited *z*-resolution in the DAPI channel (3 µm slice distance) makes 3D segmentation challenging, while Merscope's tissue-clearing process precludes subsequent imaging modalities like DNA-FISH.
2. *Resolution and data accessibility*: While all platforms provide sufficient resolution for *ST* applications, MC exhibited superior optical resolution (352 ± 50 nm) when compared to Merscope (480 ± 85 nm) and Xenium (474 ± 55 nm). Access to raw image data is essential for troubleshooting low transcript counts or high background signals. Notably, Xenium was the only system that entirely restricted access to the original transcript image data.
3. *Workflow complexity and hands-on time*: Both Xenium and MC require approximately 1.5 days for slide preparation, using straightforward protocols suitable for various tissue types. Merscope demands significantly more time (5–7 days), using complex procedures, including gel embedding, that require specialized technical expertise.
4. *Probe panel customization*: Although all platforms support custom gene panels, their implementation varies. MC offers simple 100-gene custom panels, Merscope provides the highest custom-designed panel capacity (up to 960 genes), and Xenium boasts extensive catalog panels with additional customization options.
5. *Tissue size and placement considerations*: To efficiently use resources, it is important to match tissue dimensions to platform capabilities (Additional file 2: Fig. S1). MC accommodates multiple small samples in separate areas (ideal for our MBEN case with multiple patient samples). At the same time, Merscope and Xenium offer larger continuous imaging areas better suited to extensive tissue sections. Tissue placement is most challenging with Merscope due to its confined area and unmarked round slide format.
6. *Data analysis support*: All systems provide transcript coordinates, DAPI images, and quality reports, but only Merscope and Xenium include built-in cell segmentation. Xenium also offers standard gene expression clustering, potentially reducing downstream analysis complexity.

In summary, MC offers an excellent balance of flexibility and straightforward implementation for validation experiments with smaller sample sizes. Researchers looking for larger gene panels with integrated protein detection should consider Merscope despite its more complex workflow. For large-scale studies involving multiple samples or tissue areas, Xenium provides the most efficient high-throughput solution. These factors should guide the platform choice based on specific research goals and practical constraints. Additionally, it is crucial to consider the technological advancements of commercial platforms, which may require a re-evaluation of one or more of the categories discussed above.

## Discussion

Our comparative analysis of six different *ST* methods utilized MBEN cryosections as a case study. Due to its characteristic microanatomy featuring two distinct tumor cell compartments, this entity is particularly intriguing for an *ST* analysis of the interactions between proliferation, migration, and differentiation of cancer cells [20]. Furthermore, an *ST* analysis provided significant insights into the spatial relationships among tumor subclones and proliferating tumor cells in Group 3/4 medulloblastomas, as reported in two recent studies [32, 33].

The present study provides valuable insights into the application of *ST* technologies specifically for tumor cryosections, which can differ from non-malignant tissue, as illustrated by the high local cell density observed for MBEN. Other tissue and sample types, such as mouse brain sections or FFPE samples, have unique requirements for optimizing *ST* analysis. Additionally, despite our efforts to standardize protocols and improve comparability, technical variability arises from differences in the complex experimental and data analysis workflows. Factors such as tissue handling, staining efficiency, and calibration of instrument imaging settings impact sensitivity and specificity. Consequently, these factors may influence results in an experiment-dependent manner, not necessarily reflecting platform-related differences. Moreover, *ST* technologies are rapidly evolving with continuous improvements in chemistry, experimental data acquisition, and updates to instrument-specific software. For instance, the MC 1.0 instrument will be replaced by a new system, and new chemistry versions have been consistently released for various workflows. Therefore, our study was not designed to select “the best” technology. Instead, we aim to identify key differences between methods and critical steps in the workflow that deserve consideration (Table 1). Factoring these into both experimental design and result assessment aids in choosing a specific platform.

### Including snRNA-seq data

The snRNA-seq data serve as an excellent reference for selecting the probe panel for targeted *iST* methods. The *sST* methods such as Visium, Slide-seq/Curio Seeker [34] and others can also provide this information. Based on our experience, the snRNA-seq approach is more straightforward as it utilizes established single-cell sequencing workflows, with the caveat that the data depend on the quality and quantity of the isolated nuclei used as input. Moreover, the sc/snRNA-seq data can be integrated with the *iST* data using tools like Cell2Location [35] to enhance sensitivity and resolution. New panel design tools have been released to account for platform characteristics [36]. Additionally, snRNA-seq data also serve as a reliable reference for assessing the quality and coverage of the *ST* data.

### Number of probes

With good a priori information (e.g., from snRNA-seq), even relatively small probe panels like the 10 genes used for RNAscope have already resolved the main cell types (Additional file 11: Fig. S7b). Therefore, 100 well-selected genes could be more informative than several times that number of probes from a more generic catalog panel. Furthermore, the snRNA-seq data offer an excellent reference to assess the suitability of the *iST* probe panel. The latter can be utilized to perform probe-specific subsetting, clustering, and UMAP visualization based on the snRNA-seq data, and then evaluate the quality of the resulting cell type resolution [37].

### Cell segmentation

In comparative *ST* studies, mouse brain tissue is often used as a reference. However, cancer tissues like the MBEN sections analyzed here typically have a much higher cell density, making segmentation more challenging. We have found that optimizing DAPI imaging—regarding both staining and image acquisition—can significantly enhance results. Furthermore, using Cellpose for nuclei segmentation yields robust results. Because of the high cell density in the tissue, the loss of information from transcripts outside the nuclei had no apparent negative impact on the downstream analysis in our study. In addition to Cellpose, other powerful cell and nuclei segmentation tools are available, including Mesmer [10] and Stardist [38], as well as *iST*-specific tools like Baysor [9], SCS [39], and BIDCell [40]. We also recommend testing whether optimizations in the cell segmentation process translates into improvements in downstream analysis results since this part of the workflow can become quite time-consuming. Finally, several pipelines have been published that allow for the automated preprocessing of *iST* data, such as Molkart for MC [41] and the technology-agnostic SOPA [42].

### Sensitivity

The unbiased *sST* analysis by Visium encompasses the complete transcriptome. However, its detection sensitivity and spatial resolution were inadequate to resolve the MBEN microanatomy, significantly limiting its application in our case study. This aligns with recent findings by You et al. [43] indicating that Visium’s lower spatial resolution constrained its ability to delineate fine tissue features. Other sequencing-based methods, such as Seq-Scope [44], Stereo-seq [45], Slide-seq/Curio Seeker [34], and Visium HD, offer greater subcellular spatial resolution. However, it should be noted that a sufficient quantity of RNA molecules/probes needs to be captured per area, which may necessitate spatial binning at the cost of spatial resolution. Furthermore, the sensitivity of sequencing-based spatial technologies is contingent on read depth, unlike imaging-based workflows that consistently achieve full coverage in terms of transcript numbers. Enhancements in image resolution, for example, through structured illumination microscopy (SIM) [46] or other super-resolution techniques, may mitigate crowding effects that can restrict sensitivity and/or specificity.

Overall, the sensitivity of all three automated *iST* platforms used in our study was high and very similar. For the non-amplified Merscope method, values of 50% [47] or 80% [48] have been previously reported for the detection rate with cell line samples using a custom microscope and expansion. Using snRNA-seq as a reference for our analysis of transcripts per cell (Fig. 3b, Additional file 7: S5b–d), we estimate that the average detection efficiency in our experiments was between 33 and 37%. However, it is essential to consider that a transcripts per cell analysis can be biased for *iST* methods. When specific probe panels with limited numbers of markers per cell type are used, different tissue compositions (i.e., varying abundances of the nodular and internodular compartments in our study, see Fig. 6d–e) will lead to different mean transcripts per cell, even if the sensitivity remains the same. This is especially true since the expression levels of markers for different cell types typically vary. Nonetheless, we utilized the transcripts per cell metric as a proxy for sensitivity, assuming these effects would average out when using tissue samples from different patients as we did. It should be noted that sensitivity depends on the integrity of the RNA, which is usually very good for fresh frozen material. In contrast, RNA degradation can be significant in FFPE samples, likely resulting in substantial differences between technologies, as recently reported [17].

### Specificity

Specificity depends on both probe and tissue features, making it difficult to assess this parameter quantitatively. Cook et al. [16] established spatial autocorrelation analysis as a robust metric for evaluating specificity across tissue types, a method we expanded to compare target versus background probe distributions. The background probes used in the various systems typically overlap with low signal target probes. Consequently, there is no clear cutoff regarding true negatives and false positives in terms of cumulative counts. Incorporating the spatial distribution of probes as an additional parameter to differentiate between random and more localized, genuinely specific binding is beneficial but also necessitates a probe-by-probe interpretation of the results. As demonstrated here, analyzing distribution patterns through spatial autocorrelation and next-nearest-neighbor distance analysis can offer valuable insights into probe specificity, regardless of global expression levels, which can sometimes present misleading results.

### Reimaging and including additional readouts

Reimaging the tissue enhances image quality for segmentation and/or incorporates additional readouts, benefiting from the non-destructive sample processing and slide format of MC and Xenium. The preferred method for achieving improved resolution and a higher signal-to-noise ratio is using SDCM systems equipped with highly sensitive sCMOS or EMCCD cameras. We found that imaging with point confocal microscopes was too slow for larger tissue areas. For mouse brain tissue, Hartman and Satija [19] demonstrated that better imaging and segmentation enhanced cell type resolution, which supports our findings regarding reimaging approaches. Alternatively, it is also possible to conduct an additional analysis on automated commercial widefield platforms, as shown here for the immunostaining on the Comet system following the Xenium run (Fig. 7b).

The Merscope samples are less suitable for reimaging due to their slide format and sample clearing. However, the platform integrates membrane staining into the standard workflow (a feature that also became available for other systems) and offers additional custom readouts via its auxiliary channels. The latter can be used flexibly, for instance, with signal amplification, as illustrated in Fig. 7c, or for antibody staining. Currently, alongside *ST* methods, various spatially resolved (epi)genome, proteome, and metabolome readouts are becoming available that are, in many cases, non-destructive and compatible with one another [4, 7, 49]. Thus, both commercial instruments and custom academic workflows are emerging in spatial multi-omics approaches that will further enhance the depth of insight gained from analyzing the same tissue section, as opposed to combining separate analyses of consecutive sections [4, 49, 50].

## Conclusions

In summary, by utilizing MBEN as a model system, we have developed a technology-agnostic framework applicable to diverse tissue types. This framework includes spatial autocorrelation analysis, sensitivity metrics based on snRNA-seq comparisons, and standardized resolution measurements. We find that for cryosections of tumor tissue, where RNA quality is likely less of an issue, all three automated *iST* methods performed very well regarding their sensitivity and specificity in our case study. Additionally, the spatial distribution of cell types, annotated based on the shared set of 96 genes studied, yielded very similar representations of the MBEN tissue microanatomy and cellular neighborhoods. Our findings complement several recent comparative studies of *ST* technologies across various tissue types and sample preparations. A broad comparison across six cancer types conducted by Cervilla et al. [51] reported comparable cell type identification capabilities among automated *iST* platforms, which aligns with our MBEN tissue observations. Thus, choosing one technology platform over another depends on the selection criteria discussed earlier. These criteria arise from differences in the technologies and their implementation and the associated requirements for practical work regarding both experimental and data analysis aspects. Notably, the analytical framework we developed—combining spatial statistics, segmentation quality assessment, and background probe analysis—provides metrics that can be applied across various tissue types and *ST* platforms to guide technology selection based on specific research objectives.

## Methods

### Tissue samples

The MBEN samples MB263, MB266, MB295, and MB299 used in this study have been previously described [20], and their analysis with the various technologies is detailed in Additional file 1: Table S1. Cryosections of 10 µm thickness were obtained using a Cryostar NX50 (Epredia) cryostat at a cutting temperature of −15 °C for all technologies. Subsequent storage and processing were carried out in accordance with the standard protocols provided for each workflow, as detailed below.

### snRNA-seq

The snRNA-seq data were sourced from ref. [52] and obtained using the Chromium drop-seq platform with the 3′-Single Cell RNA-sequencing v2 kit (10x Genomics).

### Visium

Tissue slices of 10 µm were placed on the Visium slides and fixed with methanol at −20 °C. After H&E staining, the samples were imaged using an Olympus VS200 scanner, and the tissue was lysed for 4 min according to the tissue optimization results obtained previously. Visium libraries were generated following the manufacturer’s recommendations. Libraries were quantified using TapeStation and Qubit and sequenced on a NovaSeq 6000 machine, pooling four libraries per lane.

### RNAscope

The RNAscope data [52] included 10 targets (Supplementary Dataset 1) and were obtained as described in ref. [20] using the RNAscope HiPlex assay (ACD/Biotechne) according to the RNAscope HiPlex Assay User Manual (324,100-UM) from the manufacturer with minor adaptations. For MB266 and MB299, four transcripts (labeled with Alexa488, Atto550, Atto647, and Alexa750 fluorescent dyes) were imaged in three rounds, while for MB295, three transcripts (Alexa488, Atto550, Atto647, and DAPI) were imaged in four rounds using the RNAscope HiPlex Alternate Display Module (R1-R4). Flatfield correction was performed based on DAPI images and used for nuclei segmentation with Cellpose as detailed below. Spot calling of transcripts was done on maximum intensity projections with RS-FISH. Called transcripts from all rounds and colors were reformatted and combined into an output file in MC format.

### Molecular Cartography

The MC data from ref. [53] were collected using the probe set provided in Supplementary Dataset 1. In brief, the tissue sections were cut at −15 °C, and 10 µm sections were placed on MC slides in their respective areas. After placement, tissue adhesion was facilitated by briefly warming the tissue with fingertips. Slides were stored at −80 °C for less than one week before processing. These data were additionally analyzed here by restaining them with DAPI and reimaging them on the Andor Dragonfly SDCM system. Image processing followed the workflow depicted in Fig. 2a, which included stitching, correction, and registration as described in greater detail in the image analysis section below. The resulting images were then processed using the resolve-processing pipeline (https://github.com/scOpenLab/resolve_processing). First, the images were enhanced using contrast limited adaptive histogram equalization (CLAHE) [54] with a kernel size of 127, a clip limit of 0.01, and 256 bins. The processed images were then segmented using Cellpose2 with the “cyto” model, applying a probability threshold of one and a cell diameter of 70. After cell segmentation, transcripts were deduplicated with MindaGap software (https://github.com/ViriatoII/MindaGap), using a tile size of 2144 and a window size of 30, accounting for shifts calculated from transcripts with fewer than 400 copies in the window and occurring at least 10 times. Finally, transcripts were assigned to cells based on their overlap with the segmentation mask and analyzed as described below.

### Merscope

Tissues were sectioned into 10 µm slices and placed on one Merscope slide. Subsequently, the tissue was fixed with 4% PFA at 37 °C for 15 min. After washing with PBS, the sections were permeabilized with 70% ethanol and stored at 4 °C until hybridization began, which was less than two weeks later. The panel (Supplementary Dataset 1) was hybridized for 48 h, and all steps were performed according to the manufacturer’s protocol, including the membrane staining.

### Xenium

Tissues were sectioned into 10 µm slices, and four samples were placed on a single Xenium slide. After sectioning, the slides were stored at −80 °C for less than 2 weeks. On the day of the experiment, the tissue was fixed with PFA according to the manufacturer’s protocol. Tissues were permeabilized with SDS, incubated in 70% ice-cold methanol, and washed with PBS. Hybridization of the human generic brain panel with 70 add-on genes (Supplementary Dataset 1) was performed at 50 °C in a Bio-Rad C1000 touch cycler for 20 h. The washing, ligation, and amplification steps were carried out according to the manufacturer’s instructions. ROIs were selected based on the tissue area, excluding non-tissue-covered tiles. Each transcript was imaged in a bright state five times across 60 cycle-channels (15 cycles × 4 channels). After the run on the Xenium analyzer, slides were removed, and buffer was exchanged with PBS-T for further storage at 4 °C.

### H&E staining

H&E staining of Visium slides was performed by first removing the coverslip through incubation in 1 × PBS Buffer, followed by washing with H_2_O. Next, slides were incubated in hematoxylin solution for 7 min and then washed with H_2_O. Following this, 300 µl of bluing solution was added to the tissue, incubated for 2 min at room temperature, and then washed with H_2_O. Staining with eosin solution (Sigma, diluted 1:10 in 0.45 M Tris acetic acid, pH6) was carried out for 1 min at room temperature, followed by washing with H_2_O. Subsequently, slides were dehydrated through a series of washes in 70% (30 s), 95% (30 s), and two washes in 100% (1 min) ethanol, before being stored at room temperature. Virtual H&E staining followed the approach described previously [30]. Sections were stained with eosin solution for 1 min at room temperature, washed in H_2_O, and incubated for 15 min in 4 × SSC (saline-sodium citrate) buffer. Sections were then stained with DAPI for 30 s and mounted in Prolong Gold Antifade (Thermo Fisher Scientific). The H&E coloring of the DAPI and eosin staining was conducted in R using the EBImage [55] along with custom scripts.

### Spinning disk confocal fluorescence microscopy

Imaging of RNAscope samples and reimaging of MC and Xenium slides via SDCM were performed on an Andor Dragonfly 505 spinning disk confocal system, which is equipped with a Nikon Ti2-E inverted microscope and either a CFI P-Fluor 40×/1.30 oil objective or a Plan Apo 60×/1.40 oil objective*.* Multicolor images were captured using laser lines of 405 nm (DAPI), 488 nm (Alexa 488, eosin), 561 nm (Atto 550), 637 nm (Atto 647), and 730 nm (Alexa 750). The images were recorded at a 16-bit depth and with dimensions of 1024 × 1024 pixels per tile (pixel size: 0.301 µm or 0.217 µm for the 40× or 60× objective, respectively) using an iXon Ultra 888 EMCCD camera. The region of interest was chosen based on the DAPI signal, and 50 *z*-slices were captured with a step size of 0.4 µm (20 µm *z*-range) for each field of view (FOV). Tiles were imaged with a 10% overlap to ensure accurate stitching. Subsequently, flatfield correction was performed based on the DAPI channel, and the stitching and registration of the tiles were conducted using Fiji as described below.

### Merscope amplification

Gene-specific probes and amplification oligonucleotides were tested using the protein verification kit provided by Vizgen for the Merscope. A list of primary, secondary, and amplification probes is available in Additional file 14: Table S6. The tissue was fixed and permeabilized as described above, washed with 30% formamide in 2 × SSC (wash buffer), and incubated with the primary probes at a 1 µM concentration in hybridization buffer (0.05% yeast rRNA, 1 U/µl RNase inhibitor, 30% formamide, 2 × SSC, 10% dextran). After 36 h of incubation at 37 °C in a humid environment, the tissue was washed three times with wash buffer at 47 °C. The tissue was embedded according to the manufacturer’s instructions and incubated in clearing solution for 24 h. Then, the tissue was washed with amplification buffer (10% formamide, 2 × SSC), and the primary amplifier was hybridized at 5 nM in hybridization buffer (0.05% yeast rRNA, 1U/µl RNase inhibitor, 10% formamide, 2 × SSC, 10% dextran) for 30 min at 37 °C. After three washes with amplification buffer, the secondary amp probe was hybridized at a 5 nM concentration in amp hybridization buffer (0.05% yeast rRNA, 1U/µl RNase inhibitor, 10% formamide, 2 × SSC, 10% dextran) for 30 min at 37 °C. Following three washes, the verification reagent was added for 15 min, followed by a formamide and sample prep wash. The readout of the amplification probe was performed with the protein verification kit (mouse, rabbit, goat), utilizing only the mouse and rabbit channels.

### Sequential IF using the Comet platform

After the Xenium run was completed, the slides were washed twice with PBS and then placed in the Comet system (Lunaphore). The immuno-oncology SPYRE panel (Additional file 13: Table S5) was used to stain and image the tissue section of sample MB299 using the standard SPYRE protocol on a Comet 1.0 instrument.

### Preprocessing of iST data for downstream analysis

For Xenium datasets (post XeniumRanger), we cropped selected areas because some tissue sections were folded, wrapped, and disrupted. This is done to eliminate potential issues in further downstream analysis steps. A custom script is available at https://github.com/alikhuseynov/add-on_R/blob/develop/R/crop_seurat_v1.R, and related discussion can be found here https://github.com/satijalab/seurat/issues/8457.

### Cell segmentation

For cell segmentation, the methods utilized in the Merscope (Cellpose2 for nuclei segmentation or cell segmentation paired with an additional cell boundary stain) and Xenium systems (nucleus segmentation using a custom neural network followed by a 15-µm Voronoi-based cell boundary expansion) were employed. For MC, cell segmentation was conducted with Cellpose2 as described above [8]. For independent segmentation of the DAPI images of nuclei and cell membrane staining, when applicable, Cellpose2 was utilized. The corresponding scripts to overlay images with the segmentation results were created using the R script BrushUpSegmentationResults.R.

### Image processing and integration with ST data

Widefield images from the MC and Xenium platforms were integrated with reimaged SDCM data using the following workflow in ImageJ. First, SDCM image stacks underwent a maximum intensity projection, followed by flat field and chromatic aberration correction using a custom script. Next, image tiles were stitched together using the “Grid/Collection Stitching” plugin. DAPI images from SDCM were registered to MC or Xenium widefield images via “Register Virtual Stack Slices” with the Affine feature extraction model and the Elastic bUnwarpJ splines registration model. In the case of further staining, images were transformed using Transform Virtual Stack Slices, employing the transformation file of the DAPI registration to all other image channels. Custom scripts are available on GitHub [56] and Zenodo [57]. Additional data analysis software included roifile [58], Pillow [59], tifffile [60], opencv-python-headless [61], QuPath [62], and sf (simple feature access) [63].

### Combining data sets

Most of the analysis and visualization (including tidyverse, data.table, ggridges R packages) was conducted in R 4.2.2 and Bioconductor 1.30.18 R packages [64]. Raw data were processed using technology-specific corporate pipelines (a custom pipeline was used for MC). For each technology, Seurat objects of the sample data and analysis results were created using the Seurat (v. 4.3.0) R package [65]. To load Vizgen/Merscope data and making a Seurat object, we optimized a loading function (see this PR https://github.com/satijalab/seurat/pull/7190), which was also separately tested by Vizgen.

MC Seurat objects were created from the ROI file, segmentation mask, deduplicated transcripts, and cell expression matrix generated with the resolve_processing pipeline (https://github.com/scOpenLab/resolve_processing, described above) using custom R scripts (https://github.com/scOpenLab/resolve-analysis). We merged technology-specific object subsets for the same matching genes (96 in total) into a single object. When comparing to RNAscope, only 10 matching genes were used. Cells with zero counts were removed. To address issues with the subset function on Seurat objects with spatial FOVs (see https://github.com/satijalab/seurat/issues/6409, https://github.com/satijalab/seurat/issues/7462), we wrote and used an optimized version, which is available on GitHub [66] and Zenodo [67].

### Analysis of transcript counts per spatial bin or cell/nucleus

The distribution of transcript and feature/gene counts was analyzed for the shared set of 96 genes (Supplementary Dataset 1). It was either based on the number of transcripts in spatial bins with the size of a Visium spot of 55 µm diameter and 2375 µm^2^ area, corresponding to a square side length of $55\mathrm\mu m/2\cdot\sqrt{\mathrm\pi}=48.74\mathrm\mu m$, or on transcripts per nucleus/cell following segmentation. The spatial binning enables an unbiased comparison at the chosen bin size, free from confounding effects related to cell/nucleus segmentation. At the gene level, we computed the mean transcript counts across all cells and compared these values among the different technologies. We performed pairwise gene expression correlation analysis (Pearson correlation) for selected markers within individual cells. The similarity to the RNAscope pattern was subsequently calculated as the coefficient of determination (*R* squared) of the correlation coefficients (Additional file 8: Table S3).

### Specificity analysis using background probes

To evaluate the specificity of *iST* methods, we used the probes included with the reagents for MC (25 false positive probes), Merscope (40 blank probes), and Xenium (128 unassigned codeword probes) (Supplementary Dataset 1), which we refer to here as background probes. The signals of 96 shared target genes and the background were related based on their coordinates in a segmentation-free manner. We determined the number of target probes overlapping with the background signal by counting the spots of a given probe per tissue and ranking the sum of the probe signals. Averaged FDR values were calculated from the same data as.

$$\mathrm{FDR}\left(\%\right)=\frac{\mathrm{background\_barcode\_calls}}{\mathrm{number\_of\_background\_barcodes}}\cdot\frac{\mathrm{number\_of\_target\_genes}}{\mathrm{total\_target\_gene\_calls}}\cdot100.$$

To evaluate the spatial distribution of target and background probes at the cellular level, the spatial autocorrelation for each probe was calculated as Moran’s *I* using the moranfast R package (C++ implementation). This function is similar to Moran *I* from the ape R package, which is faster for large datasets. The input for computing Moran’s *I* with moranfast included the transcript counts per cell and the xy coordinates of the cell centroids. The spatial neighborhood was defined using a distance-based (Euclidean) approach that calculates distances *r* between pairs of cell centroids, resulting in a distance matrix. The weighted inverse distance matrix was calculated as 1 divided by the distance matrix, meaning that the larger the resultant weight, the closer the cell centroids. This method was selected over spatial contiguity-based approaches (queen, rook, hexagon, bishop spatial neighbors) since it does not require the cell borders or polygons to touch each other. Bounds of global Moran’s *I* usually go from − 1 to + 1 (similar to Pearson correlation coefficients) [68]. A value around 0 indicates a spatially random pattern, < 0 towards − 1 indicates negative spatial autocorrelation (chessboard-like pattern), and > 0 towards 1 indicates positive spatial autocorrelation (clustered, also gradient-like patterns). The actual lower and upper bounds would depend on the spatial neighborhood network (graph) and may have extreme values [69, 70]. This approach provides the spatial autocorrelation between transcripts at a cellular resolution. Since our dataset exhibited no significant anticorrelation, only fluctuations around 0 (≥ − 0.002) as the lower limit, we utilized min–max scaled Moran’s *I* from 0 to 1 in the displayed plots.

As an alternative molecule-level approach to assessing spatial relations between the signals of a given probe, the distance to its nearest neighbor was calculated using the FNN R package with the kd-tree search algorithm. The median of the resulting distribution was then used as the minimal distance value for further analysis (Supplementary Dataset 2).

### Resolution analysis with fluorescent beads

Ultra Rainbow Fluorescent Particles (0.31 µm, Spherotec) were mixed 1:1 with 20% matrigel in PBS (v/v). Around 50 µl of this bead dilution were added to the Merscope, Xenium, or MC glass slides, and incubated for 40 min at RT. Subsequently, the liquid was aspirated, PBS was added, and the respective imaging runs were started. For confocal imaging, the beads were prepared similarly with the following differences: the beads were diluted 1:6 in 20% matrigel in PBS (v/v) and then mounted with prolong antifade.

On the resulting preprocessed images from each technology, the beads were identified as local maxima. For MC, the image was thresholded to exclude pixels with intensity < 900 and > 5000; for Xenium, only the 6 th *z* plane was used, and a threshold of 100 was applied to pixel intensities; for Merscope, a threshold of 7000 was used before a white tophat filter with a 10-pixel disk kernel was applied; for confocal imaging, a threshold of 5000 was applied to pixel intensities. The skimage.feature.corner_peaks function from scikit-image [71] was used to identify the peaks. For each peak, pixels at the corresponding coordinates were extracted for each of the *X*-, *Y*-, and *Z*-axes. These values were then employed to calculate the FWHM of the signal intensity with the scipy.signal.peak_widths function from the scipy package [72]. In Additional file 4: Fig. S2, medians are reported for the *x*- and *y*-direction separately. In the main text and Table 1, only the median of both *x* and *y* FWHM is reported. The variability of the values is reported as median absolute deviation (MAD):

$$\text{MAD}=\text{median}\left(\left|{X}_{i}-\text{median}\left(X\right)\right|\right)$$

### Signal-to-noise and signal-to-background analysis

We called spots on the primary MC images using RS-FISH to assess the quality of the primary RNA signals. Line profiles spanning ten pixels to the left and right (in *x*-direction) from the called signal spot were quantified. For normalization, the intensity of the darkest pixel of the image *I*_dark_ was subtracted from all raw intensity values *I*_raw_: *I* = *I*_raw_ − *I*_dark_. The signal-to-noise ratio SNR was then calculated as follows:

$$\text{SNR}=\frac{I\left(0\right)-{\mu }_{bg}}{{\sigma }_{bg}}$$

where *I*(0) is the peak intensity at the center, *µ*_bg_ is the mean background intensity (${\mu }_{bg}=mean\left(I\left(x\right)\right),x5\vee x>5$) and ${\sigma }_{bg}=sd\left(I\left(x\right)\right),x5\vee x>5$ is the standard deviation of the background intensities. The signal-to-background ratio (SBR) is given by:

$$\text{SBR}=\frac{I\left(0\right)}{{\mu }_{bg}}$$

### Integration, clustering and cell type annotation

We used Seurat’s SCTransform [73] and RunPCA to normalize the data. Batch correction was performed using the Harmony v1.0 [74] R package on only two samples (MB266 and 295) for each technology separately (MC, Xenium, Merscope). When integrating snRNA-seq with these three spatial technologies, batch correction was also applied to samples from the merged object (snRNA-seq, MC, Xenium, Merscope). Clustering was conducted for each technology on an integrated object using the Leiden algorithm [75], and results were visualized as UMAPs (all performed using Seurat). Cell type annotations were manually assigned based on the gene expression signatures previously reported [20].

### Neighbourhood analysis

The neighborhood analysis was conducted using a custom neighborhood_analysis R script [66, 67]. The 10 nearest neighbors of each cell were identified through the Euclidean distance between centroids, utilizing a KNN-tree in conjunction with the DBSCAN package [76]. Subsequently, the distribution of cell types within this set of neighbors was compared to a random distribution achieved by performing 10,000 permutations of the cell types assigned to each neighbor. The false discovery rate of these comparisons was managed using the Benjamini–Hochberg procedure [77].

## Supplementary Information

Additional file 1: Table S1. Number of analyzed cells per sample and technology.

Additional file 2: Fig. S1. Workflow features of different *ST* methods.

Additional file 3: Table S2. Features of different *ST* methods.

Additional file 4: Fig. S2. Resolution of different microscopy systems used in this study.

Additional file 5: Fig. S3. Signal-to-noise and Signal-to-background analysis of signal spots.

Additional file 6: Fig. S4. Quantification of different imaging and segmentation methods.

Additional file 7: Fig. S5. Sensitivity analysis of *iST* methods.

Additional file 8: Table S3. Similarity of marker gene correlation coefficient panel.

Additional file 9: Table S4. Controls and their nomenclature used in *iST*.

Additional file 10: Fig. S6. Specificity analysis from target and background probe signals.

Additional file 11: Fig. S7. Clustering and cell type annotation for different *ST* methods.

Additional file 12: Fig. S8. Clustering and cell type annotation of reimaged Xenium slides.

Additional file 13: Table S5. Antibody panel used with the COMET system.

Additional file 14: Table S6. Primary, secondary and amplification probes for Merscope.

Additional file 15: Table S7. Inventory of supplementary data sets associated with this manuscript.

Additional file 16: Table S8. Inventory of datasets deposited at external repositories.

Additional file 17: Table S9. Data analysis packages and software from external sources.

Additional file 18: Table S10. Data analysis software from this study.

Additional file 19: Supplementary Dataset 1. Probe set used for the different iST methods.

Additional file 20: Supplementary Dataset 2. Expression levels, spatial autocorrelation, and median nearest neighbor distance of panel genes and negative controls.

Additional file 21: Supplementary Dataset 3. Expression signatures and cell type annotation of MC, Merscope and Xenium data.

## Data Availability

An overview of the supplementary data associated with this manuscript is provided in Additional file 15: Table S7 and Additional file 16: Table S8. Primary data and results from downstream analyses, including those from ref. [20], can be accessed at the following locations: snRNA-seq, GEO accession number GSE239854 [52]; RNAscope, BioImage Archive accession number S-BIAD826 [78]; MC, BioImage Archive accession number S-BIAD825 [79] and GEO accession number GSE247736 [53]; additional MC data as well as Merscope, Comet, and Xenium data can be found in the BioImage Archive (accession number S-BIAD1093) [80]; Visium data and Seurat objects are available on Zenodo [81]. Data associated with the FWHM (Additional file 4: Fig. S2), signal-to-noise and signal-to-background analysis (Additional file 5: Fig. S3) are also available on Zenodo [81]. The data analysis software utilized is listed in Additional file 17: Table S9 and Additional file 18: Table S10. Custom analysis software tools can be accessed via the GitHub repositories [56, 66] and Zenodo [57, 67].
