# Supplementary material for: Comparison of spatial transcriptomics technologies using tumor cryosections
Source: Genome Biol. 2025 Jun 20;26:176. doi: 10.1186/s13059-025-03624-4 (PMC12180266; doi:10.1186/s13059-025-03624-4)
Supplement: Supplementary file 1 — Additional file 1: Table S1. Number of analyzed cells per sample and technology. [file 13059_2025_3624_MOESM1_ESM.pdf]

Table S1. Number of analyzed cells per sample and technology.

Sample ID	SnRNA-seq	RNAscope	Visium[a)]	MC	Merscope	Xenium
MB263	2701	–	1977	32106	–	83174
MB266	2246	25977	2782	34625	61514	125056
MB295	3726	72700	2490	40816	78402	181935
MB299	5009	48677	2660	30962	–	262161

Each sample listed was run once on the indicated platform.

[a] For Visium, the number corresponds to the spots under the tissue with each spot comprising RNA from several cells due to the limited resolution.
