# Supplementary material for: Comparison of spatial transcriptomics technologies using tumor cryosections
Source: Genome Biol. 2025 Jun 20;26:176. doi: 10.1186/s13059-025-03624-4 (PMC12180266; doi:10.1186/s13059-025-03624-4)
Supplement: Supplementary file 2 — Additional file 2: Fig. S1. Workflow features of different ST methods. [file 13059_2025_3624_MOESM2_ESM.pdf]

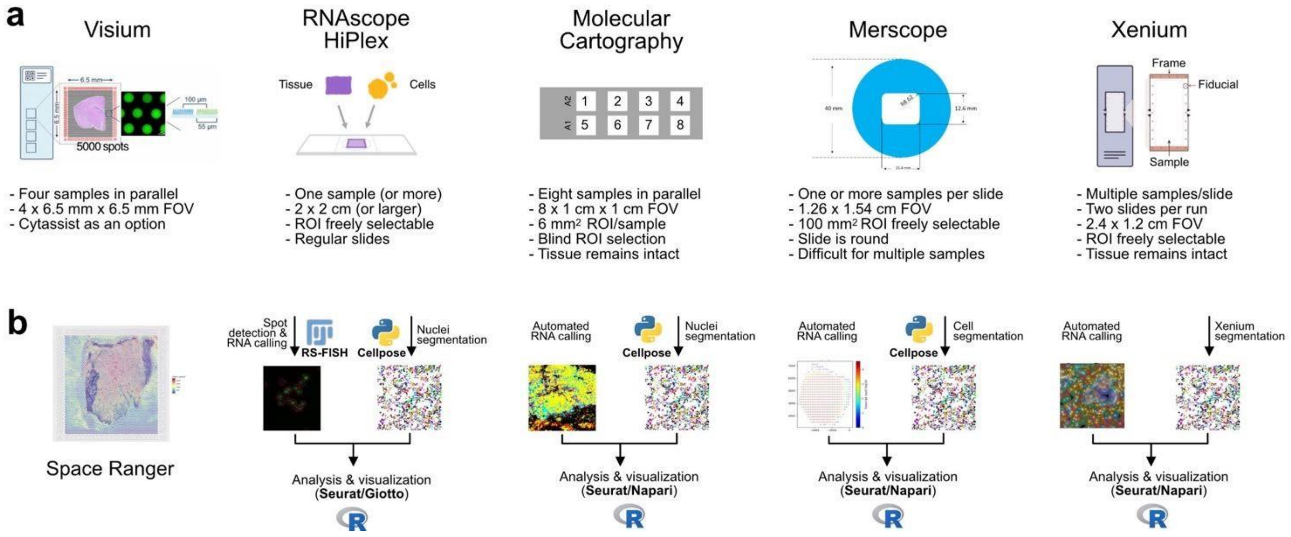

Fig. S1. Workflow features of different *ST* methods

(**a**) Slide format and features. (**b**) Scheme of data analysis workflow used in the present study.
