# Supplementary material for: Comparison of spatial transcriptomics technologies using tumor cryosections
Source: Genome Biol. 2025 Jun 20;26:176. doi: 10.1186/s13059-025-03624-4 (PMC12180266; doi:10.1186/s13059-025-03624-4)
Supplement: Supplementary file 3 — Additional file 3: Table S2. Features of different ST methods. [file 13059_2025_3624_MOESM3_ESM.pdf]

Table S2. Features of different *ST* methods.

	Visium	RNAscope HiPlex	MC	Merscope	Xenium
**Instrument requirements**	DNA sequencer	Fluorescence microscope	MC 1.0	Merscope	Xenium Analyzer
**Resolution**	~100 µm	Subcellular	Subcellular	Subcellular	Subcellular
**Gene panel number**	Unbiased	10 (shared)	100 (96 shared with Merscope & Xenium)	138 (96 shared with MC)	345 (96 shared with MC)
**Amplification**	PCR	~100-1000 fluorophores per target	Direct labeling of RNA locus with >20 probes	Direct labeling of RNA locus with ~30-50 probes	Rolling circle amplification of padlock probes
**Transcript assignment**	3'-sequencing & genome mapping	Fluorophore color	Combinatorial decoding in 8 imaging rounds	Combinatorial decoding in 15 imaging rounds	Combinatorial decoding in 18 imaging rounds
**Controls**	–	Negative control targeting bacterial RNA	28 FP (negative control codewords)	40 blank (negative control codewords)	128/41/20 unassigned /negative codewords/ negative probes
**H&E staining**	Before run	Before/after run[a]	After run	Not compatible	After run

[a] We performed virtual H&E staining using DAPI and eosin (see Methods for details) prior to RNAscope HiPlex. A tissue permeabilization step with protease in the protocol renders the tissue unsuitable for subsequent H&E staining. Newer RNAscope protocols utilize alternatives for tissue permeabilization and are compatible with H&E and immunostaining.
