# Supplementary material for: Comparison of spatial transcriptomics technologies using tumor cryosections
Source: Genome Biol. 2025 Jun 20;26:176. doi: 10.1186/s13059-025-03624-4 (PMC12180266; doi:10.1186/s13059-025-03624-4)
Supplement: Supplementary file 4 — Additional file 4: Fig. S2. Resolution of different microscopy systems used in this study. [file 13059_2025_3624_MOESM4_ESM.pdf]

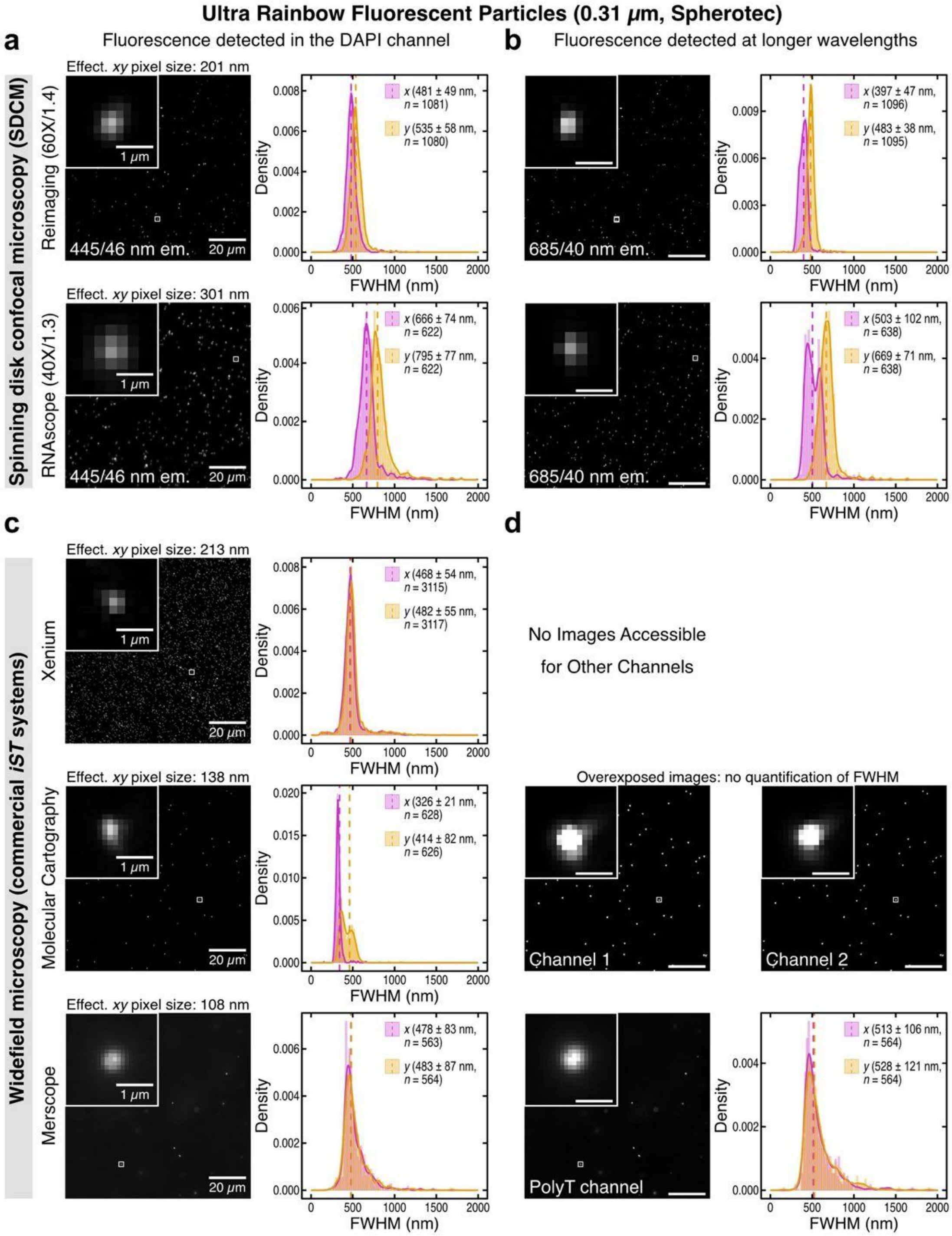

Fig. S2. Resolution of different microscopy systems utilized in this study

Multicolor fluorescent particles with a diameter of 0.31 µm were imaged across all systems, and the FWHM was measured. Values reported here are medians ± median absolute deviation (see Methods for details). (**a**) Bead images and FWHM for the DAPI channel (emission at 445/46 nm) using the 40x objective in RNAscope HiPlex and the 60x objective for reimaging Xenium and MC slides. The FWHM for the 40x confocal was unusually high (~670-800 nm), primarily due to the large effective pixel size of the

EMCCD camera, which has an effective pixel size of 301 nm in the image plane. This results in the undersampling of particles. The resulting broadening of spots would affect the detection of crowded transcripts in RNAscope HiPlex but would only impact single genes since RNAscope HiPlex does not rely on combinatorial barcoding. (**b**) Same as panel **a** for the red channel (emission at 685/40 nm). (**c**) Same as panel **a,** but for the commercial widefield *iST* systems analyzed here. (**d**) Same as panel **c** for other channels where image data was available. Information on emission filters and fluorophores is not provided.
