# Supplementary material for: Comparison of spatial transcriptomics technologies using tumor cryosections
Source: Genome Biol. 2025 Jun 20;26:176. doi: 10.1186/s13059-025-03624-4 (PMC12180266; doi:10.1186/s13059-025-03624-4)
Supplement: Supplementary file 5 — Additional file 5: Fig. S3. Signal-to-noise and Signal-to-background analysis of signal spots. [file 13059_2025_3624_MOESM5_ESM.pdf]

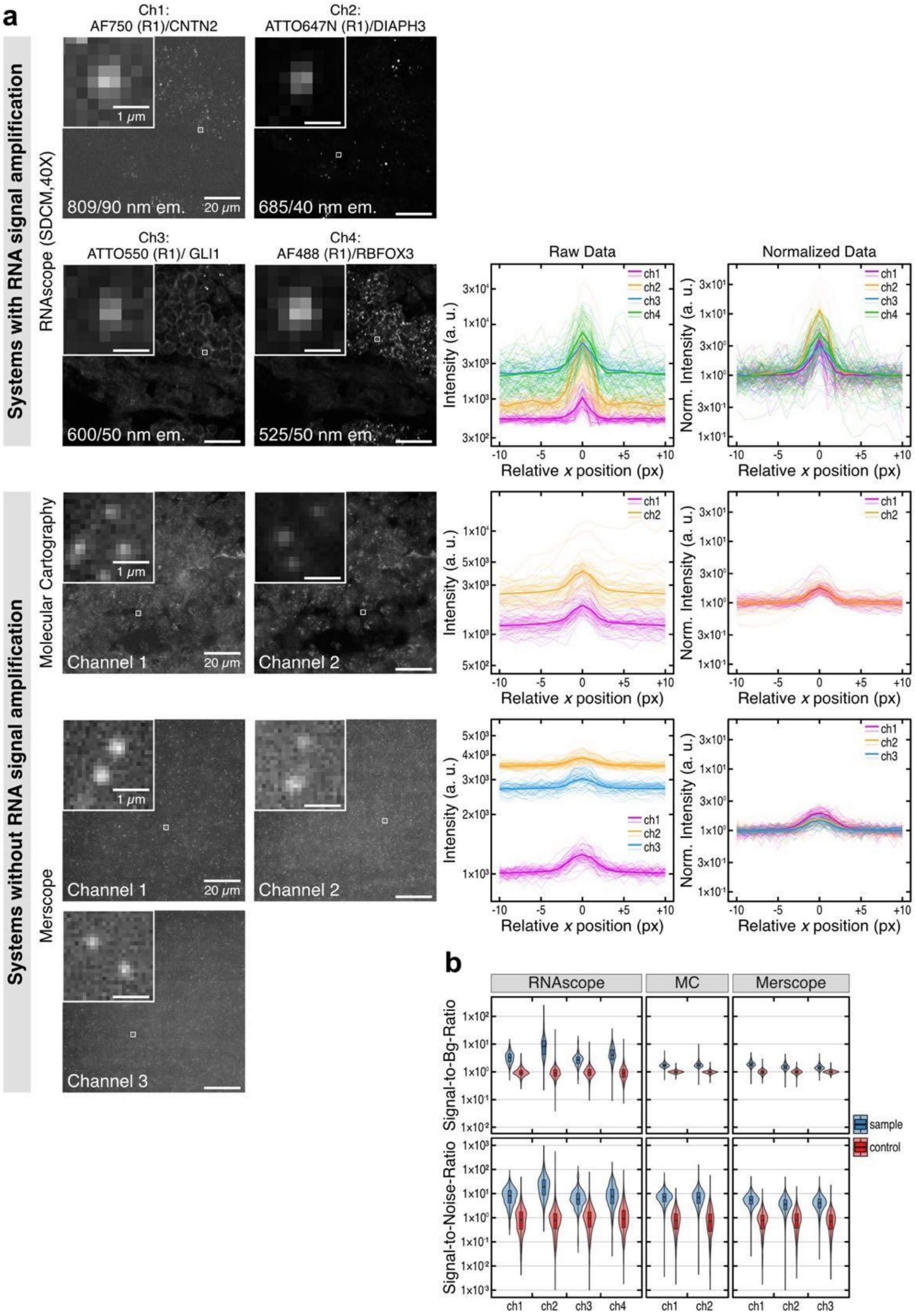


**Fig. S3. Signal-to-noise and signal-to-background analysis of signal spots**

Spots in the original images were called by RS-FISH for all methods. Then, the pixel intensities spanning 10 px on either side of the identified spot in the *x*-direction were analyzed. (**a**) Exemplary RNA images for RNAscope (four colors), MC (two colors), and Merscope (three colors) are presented alongside the raw

data and normalized line profiles. The mean profiles are shown as bold solid lines, while fifty randomly selected single traces are depicted as thin, transparent lines. (**b**) Quantification of signal-to-background (SBR) and signal-tonoise ratios (SNR) is also included. SBR corresponds to the peak height (at $x = 0$) in the normalized plots from panel **a**, while SNR represents the ratio of the peak height to the standard deviation (i.e., noise) in the neighborhood of the peak (evaluated for each single profile at $x < -5$ and $x > 5$). Controls consist of line profiles from 1,000 randomly selected pixels per condition (see Methods for details).
