# Supplementary material for: Comparison of spatial transcriptomics technologies using tumor cryosections
Source: Genome Biol. 2025 Jun 20;26:176. doi: 10.1186/s13059-025-03624-4 (PMC12180266; doi:10.1186/s13059-025-03624-4)
Supplement: Supplementary file 6 — Additional file 6: Fig. S4. Quantification of different imaging and segmentation methods. [file 13059_2025_3624_MOESM6_ESM.pdf]

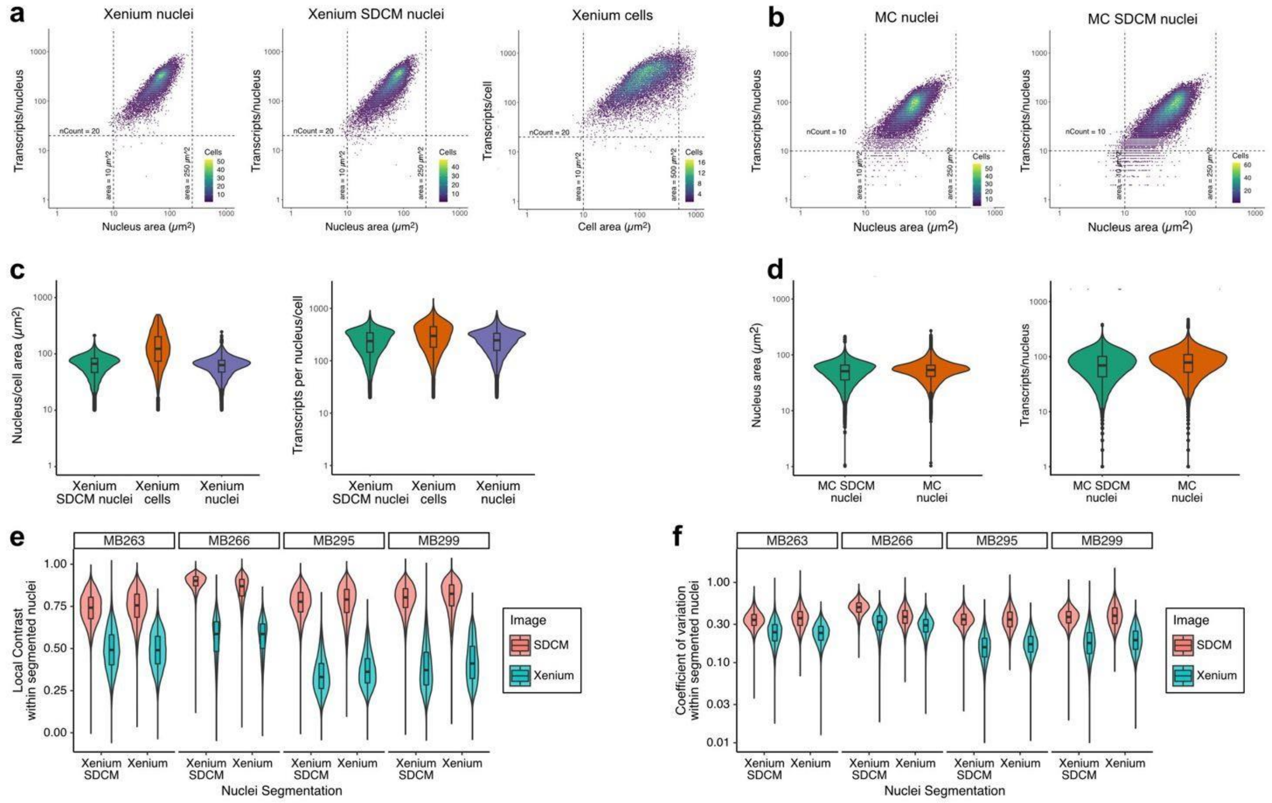

Fig. S4. Quantification of different imaging and segmentation methods

(**a**) Quantification of the number of transcripts per nucleus/cell versus nucleus/cell area. The Xenium slide for an exemplary tumor tissue (MB266) was reimaged using SDCM (Xenium SDCM nuclei). The segmentation results were compared for the number of transcripts per nucleus/cell between (i) the original widefield images acquired with the Xenium analyzer through segmentation of nuclei with Cellpose (Xenium nuclei), (ii) the Xenium segmentation workflow with the expansion of the nuclei designed to cover whole cells (Xenium cell), and (iii) the reimaged SDCM nuclei segmented by Cellpose. (**b**) Same as panel **a** but for Molecular Cartography (MC). (**c**) Violin plots displaying the size distribution of segmented nuclei/cells (left) and transcripts per segmented nucleus/cell for MB266. SDCM refers to the spinning disk confocal images compared to widefield images obtained by the Xenium analyzer. (**d**) Same as panel **c** but for Molecular Cartography. (**e**) Local or Michelson contrast calculated as (max-min)/(max+min) within segmented nuclei for the original widefield Xenium DAPI image or reimaged by SDCM. (**f**) Same as panel **e** but using the coefficient of variation (standard deviation/mean) instead of local contrast as a measure for the contrast/information content of nuclei. Regardless of which segmentation method was used for nuclei, the local contrast and coefficient of variation were consistently higher for SDCM than for widefield images, likely due to the blocking of out-of-focus light in confocal microscopy. This could explain the observed higher segmentation quality in SDCM images despite the lack of difference in resolution (**Fig. S2**).
