# Supplementary material for: Comparison of spatial transcriptomics technologies using tumor cryosections
Source: Genome Biol. 2025 Jun 20;26:176. doi: 10.1186/s13059-025-03624-4 (PMC12180266; doi:10.1186/s13059-025-03624-4)
Supplement: Supplementary file 7 — Additional file 7: Fig. S5. Sensitivity analysis of iST methods. [file 13059_2025_3624_MOESM7_ESM.pdf]

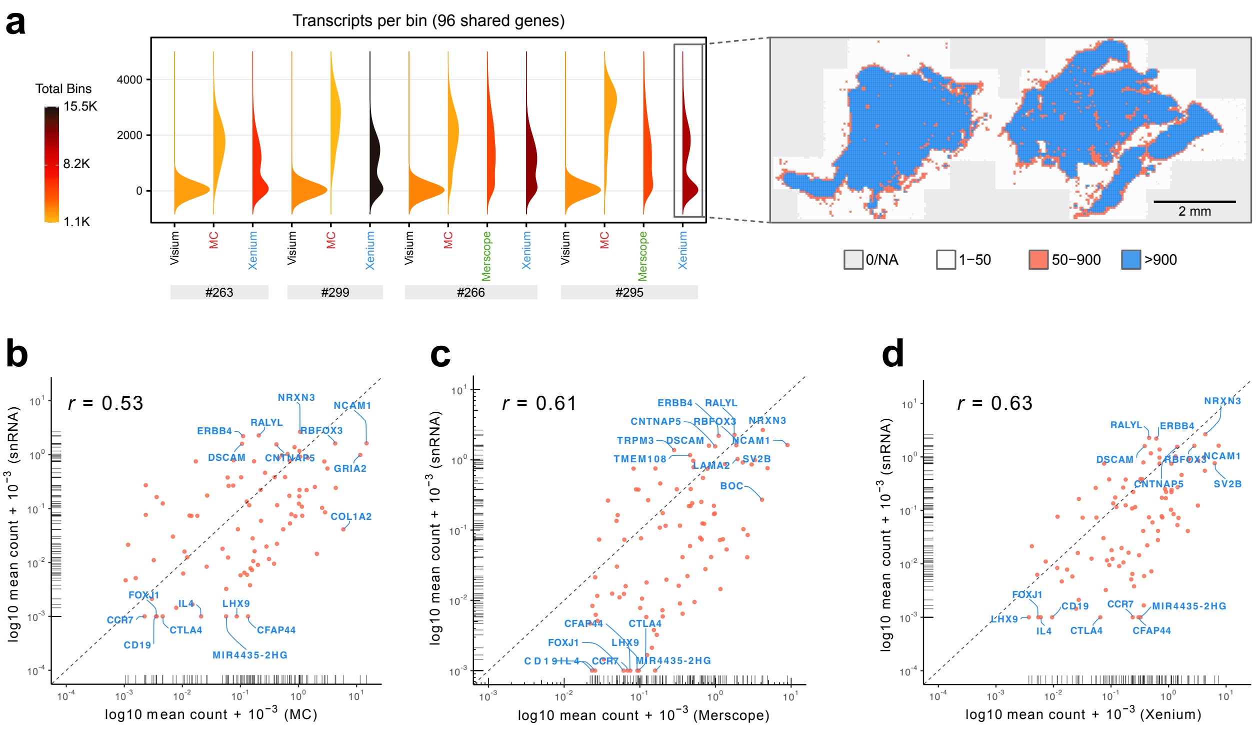

Fig. S5. Sensitivity analysis of *iST* methods

(**a**) Tissue location of cells with high and low transcript distribution for the spatial binning analysis shown in **Fig. 3a** for sample MB295 on Xenium. The cell population with fewer transcripts per bin is enriched in the outer regions of the tissue. (**b**) Correlation of snRNA-seq with MC for the shared panel of 96 genes in snRNA-seq. The dashed line indicates the equivalent number of transcripts detected by the two methods being compared. The snRNA-seq analysis was conducted using the Chromium v2 chemistry, which has a reported detection efficiency of 14-15% (v3 chemistry 30-32%) according to the manufacturer (https://kb.10xgenomics.com/hc/en-us/articles/360001539051-What-fraction-of-mRNA-transcripts-arecaptured-per-cell). (**c**) Same as panel **b,** but for the correlation of snRNA-seq with Merscope. (**d**) Same as panel **b**, but for the correlation of snRNA-seq with Xenium.
