# Supplementary material for: Comparison of spatial transcriptomics technologies using tumor cryosections
Source: Genome Biol. 2025 Jun 20;26:176. doi: 10.1186/s13059-025-03624-4 (PMC12180266; doi:10.1186/s13059-025-03624-4)
Supplement: Supplementary file 8 — Additional file 8: Table S3. Similarity of marker gene correlation coefficient panel. [file 13059_2025_3624_MOESM8_ESM.pdf]

Table S3. Similarity of correlation coefficients for marker gene panel.

	MC	Merscope	Xenium	snRNA
**RNAScope**	0.451	0.716	0.583	0.417
**MC**		0.650	0.769	0.470
**Merscope**			0.763	0.441
**Xenium**				0.707

The correlation coefficients calculated for the combinations of the 10 marker genes depicted in **Fig. 4** were compared across different technologies. The similarity was determined as R-squared values of the correlation coefficients.
