# Supplementary material for: Comparison of spatial transcriptomics technologies using tumor cryosections
Source: Genome Biol. 2025 Jun 20;26:176. doi: 10.1186/s13059-025-03624-4 (PMC12180266; doi:10.1186/s13059-025-03624-4)
Supplement: Supplementary file 9 — Additional file 9: Table S4. Controls and their nomenclature used in iST. [file 13059_2025_3624_MOESM9_ESM.pdf]

Table S4. Controls and their nomenclature used in *iST*.

Control	Origin	MC	Merscope	Xenium[d]	This study
**Secondary probe with code not in specific panel[a]**	Unspecific binding of fluorescently labeled secondary read-out probes	False positive	Blank	Unassigned codeword	Background probe
**Unspecific primary probe[b]**	Unspecific binding of random, non-targeting probe	Not used	Not used	Negative control probe	Negative control probe
**Unused decoding signal[c]**	Readout error that leads to an optical barcode that does not match that of a target gene	Not evaluated	Not evaluated	Negative control codeword	Not used

The different technology platforms use various control probe types, the nomenclature and meaning of which are given in the table.

[a] The fluorescently labeled secondary probes with code sequences not present in the primary probe panel that target specific RNAs are referred to as “false positives" for MC, "blanks" (Merscope), or “unassigned codewords" (Xenium). Here, they are designated as background probes, which represent false-positive signals. The signals obtained with these probes may overlap with those of weakly expressed genes.

[b] The Xenium system utilizes additional controls with nonspecific primary probes. Due to its amplification of the padlock probe signal, a single nonspecifically bound probe can produce a targetlike signal. With these controls, a normalized negative control probe count p_{neg} is calculated as p_{neg} $= p_{neg,total}/n_{neg}/n_{cells},$ where $p_{neg,total}$ is the total number of all negative control probe counts, n_{neg} is the number of negative control probes, and n_{cells} is the number of cells. This estimated number of false positive transcripts per cell is then calculated as $p_{neg} \times n_{target},$ where n_{target} is the number of target genes. Negative control probes are unnecessary for the MC and Merscope since they employ 2050 different probes per target. In this case, the nonspecific binding of a single primary probe would only result in mistargeting of one or two fluorophores. Therefore, it would not be detectable unless the nonspecific binding occurred at repetitive sequences.

[c] Target genes are identified from the acquired optical barcodes, which consist of specific patterns of fluorescence signals in various colors generated by multiple rounds of secondary probe hybridization and imaging. Optical barcodes that do not match those of a target indicate errors in the readout of fluorescence signals during image acquisition.

[d] In the Xenium system, a quality score Q is assigned to each decoded transcript to evaluate the confidence in its identity. The Q value is calculated based on the likelihood of the maximum likelihood codeword (i.e., the codeword that best explains the observed data)

compared to the likelihood of other suboptimal codewords. Any target with a mean *Q*-score below 20 (Q20) is excluded from further analysis.
