# Supplementary material for: Comparison of spatial transcriptomics technologies using tumor cryosections
Source: Genome Biol. 2025 Jun 20;26:176. doi: 10.1186/s13059-025-03624-4 (PMC12180266; doi:10.1186/s13059-025-03624-4)
Supplement: Supplementary file 10 — Additional file 10: Fig. S6. Specificity analysis from target and background probe signals. [file 13059_2025_3624_MOESM10_ESM.pdf]

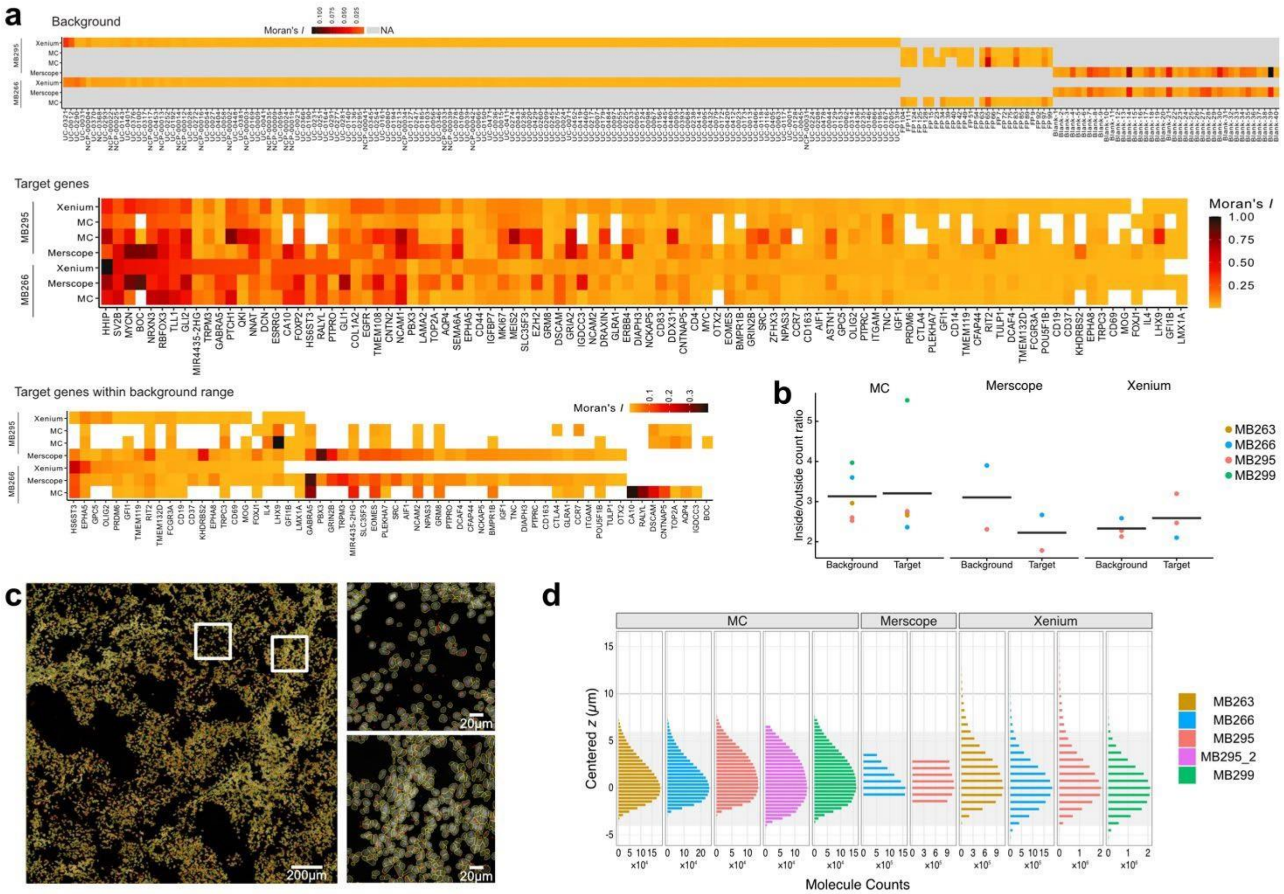

Fig. S6. Specificity analysis from target and background probe signals

The signal and spatial distribution of probes targeting the shared 96 RNAs (referred to as “target”) were compared to the nonspecific background signal. The corresponding control probes are labeled as "false positive” (MC), “blank” (Merscope), or “unassigned codeword," and these are collectively termed as “background” (see Supplementary Table S4). (**a**) Spatial autocorrelation, computed as Moran’s *I* for background probes, target genes, and target genes within background probes (*I* is min-max scaled, ranging from 0 to 1), shows a *p*-value of ≤ 0.05. A random and/or negative spatial autocorrelation approaches ~0.002 and 0. Abbreviations for Xenium background probes are detailed in **Table S4**. UC, unassigned codeword; NCP, negative control probe. (**b**) Ratios of signal counts for target and background probes located inside or outside of nuclei across different *iST* platforms. Larger ratios indicate a higher concentration of signal within the nuclei. (**c**) SDCM DAPI image of the Xenium MB266 slide overlaid with nuclei segmentation (yellow) and the background panel probes (red). (**d**) Distribution of RNA spots detected across the images recorded along the optical axis of the microscope (*z*-levels) for the different *iST* platforms. The shaded grey area represents the section thickness (10 µm). Note, however, that this is the cutting thickness, and fixation and other treatments such as gel embedding and tissue clearance in the case of Merscope will change the physical section thickness during imaging.
