# Supplementary material for: Comparison of spatial transcriptomics technologies using tumor cryosections
Source: Genome Biol. 2025 Jun 20;26:176. doi: 10.1186/s13059-025-03624-4 (PMC12180266; doi:10.1186/s13059-025-03624-4)
Supplement: Supplementary file 11 — Additional file 11: Fig. S7. Clustering and cell type annotation for different ST methods. [file 13059_2025_3624_MOESM11_ESM.pdf]

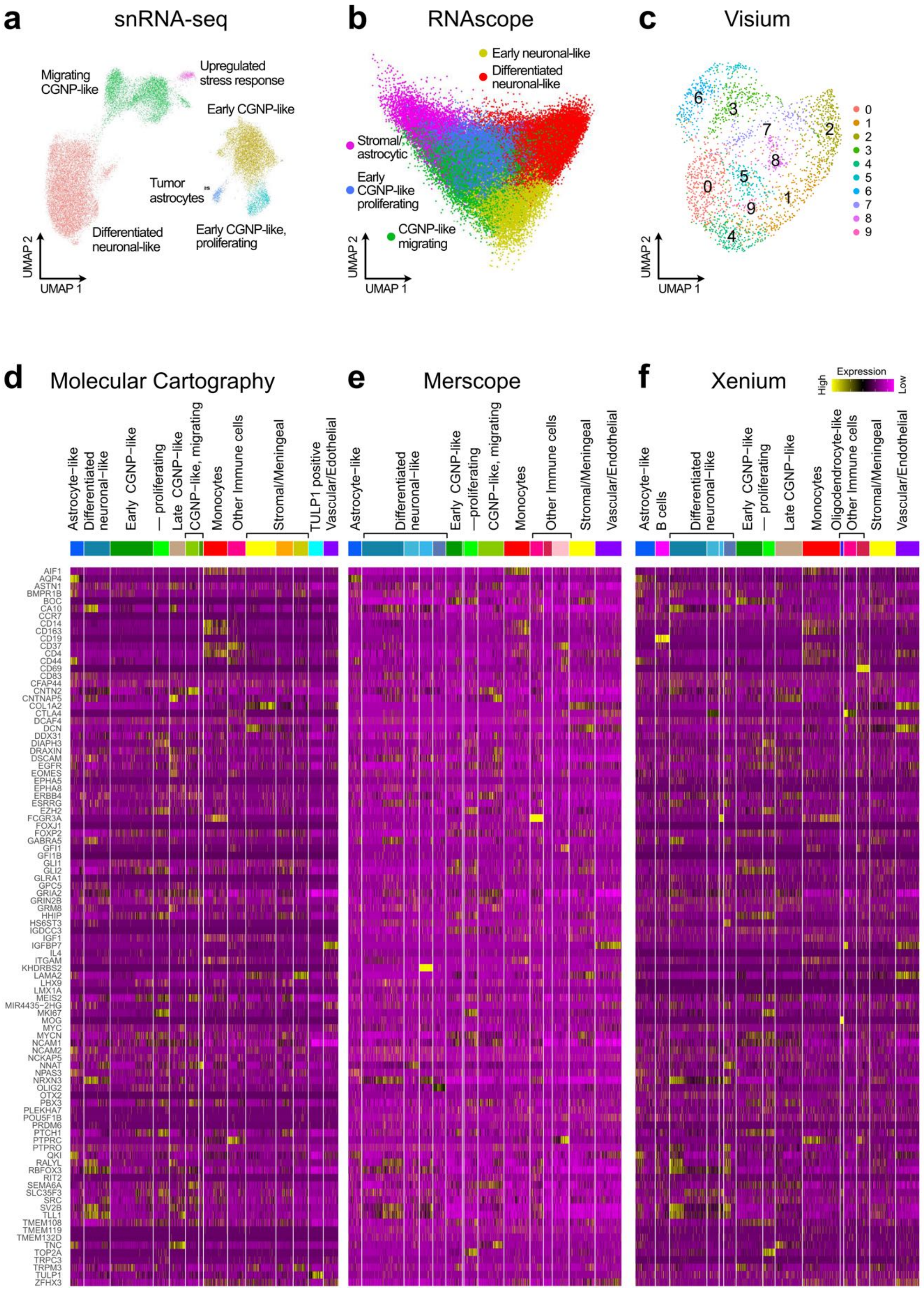

Fig. S7. Clustering and cell type annotation for different *ST* methods

Clustering was performed with the panel of 96 shared genes. (**a**) Clustering of scRNA-seq data for 27,782 cells taken from Ghasemi et al., Nat Commun 2024, 15:269. (**b**) Clustering of RNAscope data for 110,508 cells obtained from Ghasemi et al., Nat Commun 2024, 15:269. (**c**) Clustering of Visium data. Due to insufficient spatial resolution, no cell types were assigned to the identified clusters. (**d**) Heatmaps for clustering and cell type annotation of MC data. (**e**) Same as panel **d** but for Merscope. (**f**) Same as panel **d** but for Xenium.
