# Supplementary material for: Comparison of spatial transcriptomics technologies using tumor cryosections
Source: Genome Biol. 2025 Jun 20;26:176. doi: 10.1186/s13059-025-03624-4 (PMC12180266; doi:10.1186/s13059-025-03624-4)
Supplement: Supplementary file 13 — Additional file 13: Table S5. Antibody panel used with the COMET system. [file 13059_2025_3624_MOESM13_ESM.pdf]

**Table S5. Antibody panel used with the COMET system.**

| Marker | Product code | Clone | Isotype | Host species | Recommended working conc. | | |
|---|---|---|---|---|---|---|---|
| | | | | | in µg/ml | stock | Dilution |
| αSMA | MR10100 | 1A4 | IgG/k | Mouse | 0.05 | 6.3 | 118 |
| CD3 | MR10010 | LUN3 | IgG1 | Rabbit | 0.14 | 241 | 1673 |
| CD4 | MR10020 | BL-155-1C11 | IgG | Rabbit | 10.00 | 1000 | 100 |
| CD8 | MR10030 | C8/144B | IgG1 | Mouse | 1.25 | 250 | 200 |
| CD11c | MR10070 | BLR138H | IgG | Rabbit | 0.67 | 100 | 150 |
| CD20 | MR10050 | L26 | IgG2a/k | Mouse | 0.11 | 35 | 312.5 |
| CD45 | MR10090 | PD7/26 + 2B11 | IgG1/k | Mouse | 7.71 | 993 | 128 |
| CD56 | MR10060 | LUN56 | IgG1 | Rabbit | 0.13 | 33 | 250 |
| CD68 | MR10080 | KP1 | IgG1 | Mouse | 0.20 | 50 | 250 |
| FOXP3 | MR10040 | 236A/E7 | IgG1 | Mouse | 19.10 | 955 | 50 |
| Ki-67 | MR10110 | BLR021E | IgG | Rabbit | 0.07 | 50 | 700 |
| PD-1 | MR10120 | EPR4877(2) | IgG | Rabbit | 2.50 | 2002 | 800 |
| PD-L1 | MR10130 | 73-10 | IgG | Rabbit | 0.14 | 115 | 800 |
