# Supplementary material for: Comparison of spatial transcriptomics technologies using tumor cryosections
Source: Genome Biol. 2025 Jun 20;26:176. doi: 10.1186/s13059-025-03624-4 (PMC12180266; doi:10.1186/s13059-025-03624-4)
Supplement: Supplementary file 14 — Additional file 14: Table S6. Primary, secondary and amplification probes for Merscope. [file 13059_2025_3624_MOESM14_ESM.pdf]

Table S6. Primary, secondary and amplification probes for Merscope.

Target	Sequence
NES_primary_a	TGGGAGATTGAAGGTAGTGTTTCTTGAGGGGGTGGCCTCTGCTCTCCAGTGGTTAGAGTGAGTAGTAGTGGAGT
NES_primary_b	TGGGAGATTGAAGGTAGTGTTTTCTTCTTCTAGAGTCTTCAGTGGCTCCTGGTTAGAGTGAGTAGTAGTGGAGT
pre_amp_a_channel_2	ACCCATTACTCCATTACCATATACCCATTACTCCATTACCATATACCCATTACTCCATTACCATATACCCATTACTCCATTACCATATACCCATTACTCCATTACCATATACTCCACTACTACTCACTCT
pre_amp_b_channel_2	ACACTACCTTCAATCTCCCAAAACCCATTACTCCATTACCATATACCCATTACTCCATTACCATATACCCATTACTCCATTACCATATACCCATTACTCCATTACCATATACCCATTACTCCATTACCAT
sec_amp_channel_2	TTGTAAATGGAGGTGGTATATTGTAAATGGAGGTGGTATATTGTAAATGGAGGTGGTATATTGTAAATGGAGGTGGTATAATGGTAATGGAGTAATGGGT
