# Supplementary material for: Comparison of spatial transcriptomics technologies using tumor cryosections
Source: Genome Biol. 2025 Jun 20;26:176. doi: 10.1186/s13059-025-03624-4 (PMC12180266; doi:10.1186/s13059-025-03624-4)
Supplement: Supplementary file 15 — Additional file 15: Table S7. Inventory of supplementary data sets associated with this manuscript. [file 13059_2025_3624_MOESM15_ESM.pdf]

Table S7. Inventory of supplementary datasets associated with this study.

File Name	Figure/Table	Description
Supplementary Dataset 1: SuppDataset01.xlsx	Fig. 1	Probe set used for the different *iST* methods.
Supplementary Dataset 2: SuppDataset02.xlsx	Fig. 5	Expression levels, spatial autocorrelation, and median nearest neighbor distance of panel genes and negative controls.
Supplementary Dataset 3: SuppDataset03.xlsx	Fig. 6, Fig. S7, Fig. S8	Expression signatures and cell type annotation of MC, Merscope and Xenium data.
