# Supplementary material for: Comparison of spatial transcriptomics technologies using tumor cryosections
Source: Genome Biol. 2025 Jun 20;26:176. doi: 10.1186/s13059-025-03624-4 (PMC12180266; doi:10.1186/s13059-025-03624-4)
Supplement: Supplementary file 16 — Additional file 16: Table S8. Inventory of datasets deposited at external repositories. [file 13059_2025_3624_MOESM16_ESM.pdf]

Table S8. Inventory of datasets deposited at external repositories.

Data	Repository	Link	Description
snRNA-seq count tables	GEO, GSE239854	https://www.ncbi.nlm.nih.gov/geo/query/acc.cgi?acc=GSE239854	Data for samples MB266, MB295, MB299 from ref. [1].
MC transcript count tables	GEO, GSE247736	https://www.ncbi.nlm.nih.gov/geo/query/acc.cgi?acc=GSE247736	Data for samples MB263, MB266, MB295, MB299 from ref. [1]
RNAscope	BioImage Archive, S-BIAD826	https://www.ebi.ac.uk/biostudies/bioimages/studies/S-BIAD826	Raw and processed data from ref. [1]
MC (Molecular Cartography)	BioImage Archive, S-BIAD825	https://www.ebi.ac.uk/biostudies/bioimages/studies/S-BIAD825?query=S-BIAD825	Raw and processed data for samples MB263, MB266, MB295, MB299 from ref. [1]
Visium	Zenodo	https://doi.org/10.5281/zenodo.10863259	Raw and filtered feature bc matrix and spatial data for samples MB263, MB266, MB295, MB299, this study.
MC (additional data), Merscope, Xenium	BioImage Archive, S-BIAD1093	https://www.ebi.ac.uk/biostudies/bioimages/studies/S-BIAD1093?query=S-BIAD1093	Raw and processed data for samples MB263, MB266, MB295, MB299, this study.
Seurat objects for cell-based analysis	Zenodo	https://doi.org/10.5281/zenodo.10863259	Seurat objects for cell-based analysis of shared set of 96 genes for snRNA-seq, Merscope, MC and Xenium for and for shared set of 10 genes when including RNAscope, this study.
Seurat objects for segmentation-free analysis	Zenodo	https://doi.org/10.5281/zenodo.10863259	Seurat objects for segmentation-free analysis of shared set of 96 genes for snRNA-seq, Merscope, MC and Xenium for and for shared set of 10 genes when including RNAscope, this study.
Data for image analysis with fluorescent beads	Zenodo	https://doi.org/10.5281/zenodo.10863259	Data associated with the FWHM, signal-tonoise and signal-to-background analysis
