# Supplementary material for: Comparison of spatial transcriptomics technologies using tumor cryosections
Source: Genome Biol. 2025 Jun 20;26:176. doi: 10.1186/s13059-025-03624-4 (PMC12180266; doi:10.1186/s13059-025-03624-4)
Supplement: Supplementary file 17 — Additional file 17: Table S9. Data analysis packages and software from external sources. [file 13059_2025_3624_MOESM17_ESM.pdf]

Table S9. Data analysis packages and software from external sources.

Software	Ref.	Link	Version
Bioconductor	[1]	www.bioconductor.org	1.30.18
Seurat	[2]	satijalab.org/seurat/	4.3.0.9002
Cellpose 2	[3]	https://pypi.org/project/cellpose/	2.2
Voyager	[4]	https://pachterlab.github.io/voyager/	
Harmony	[5]	https://cran.r-project.org/package=harmony	1.0
SCTransform	[6]	https://cran.r-project.org/package=sctransform	
roifile	[7]	https://pypi.org/project/roifile/	2023.2.12
Pillow	[8]	https://python-pillow.org/	9.4.0
tifffile	[9]	https://pypi.org/project/tifffile/	v2023.3.15
scikit-image	[10]	https://scikit-image.org/	0.2.0
opencv-python-headless	[11]	https://pypi.org/project/opencv-python/	4.7.0.72
QuPath	[12]	https://qupath.github.io/	0.5.0
sf (simple feature access)	[13]	https://r-spatial.github.io/sf/	1.0-15
RImageJROI		https://cran.r-project.org/package=RImageJROI	0.1.2
bUnwarpJ in Fiji	[14]	https://imagej.net/plugins/bunwarpj/	2.6.13
geojsonsf		https://cran.r-project.org/package=geojsonsf	2.0.3
moranfast		https://github.com/mcooper/moranfast	
DBSCAN	[15]	https://cran.r-project.org/package=dbscan	1.1.12
scipy	[16]	https://docs.scipy.org/doc/scipy/index.html	1.15.1

References

1. Gentleman RC, Carey VJ, Bates DM, Bolstad B, Dettling M, Dudoit S, Ellis B, Gautier L, Ge Y, Gentry J, et al: **Bioconductor: open software development for computational biology and bioinformatics.** *Genome Biol* 2004, **5:**R80.

2. Stuart T, Butler A, Hoffman P, Hafemeister C, Papalexi E, Mauck WM, 3rd, Hao Y, Stoeckius M, Smibert P, Satija R: **Comprehensive Integration of Single-Cell Data.** *Cell* 2019, **177:**1888-1902 e1821.

3. Pachitariu M, Stringer C: **Cellpose 2.0: how to train your own model.** *Nat Methods* 2022, **19:**1634-1641.

4. Moses L, Einarsson PH, Jackson K, Luebbert L, Booeshaghi AS, Antonsson S, Bray N, Melsted P, Pachter L: **Voyager: exploratory single-cell genomics data analysis with geospatial statistics.** *bioRxiv* 2023**:**2023.2007.2020.549945.

5. Korsunsky I, Millard N, Fan J, Slowikowski K, Zhang F, Wei K, Baglaenko Y, Brenner M, Loh PR, Raychaudhuri S: **Fast, sensitive and accurate integration of single-cell data with Harmony.** *Nat Methods* 2019, **16:**1289-1296.

6. Choudhary S, Satija R: **Comparison and evaluation of statistical error models for scRNA-seq.** *Genome Biol* 2022, **23:**27.

7. Gohlke C: **cgohlke/roifile: v2023.2.12.** *Zenodo* 2023**:**doi: 10.5281/zenodo.7633998.

8. Murray A, Kemenade Hv, wiredfool, Clark JA, Alexander Karpinsky, Baranovič O, Gohlke C, Dufresne J, DWesl, Schmidt D, et al: **python-pillow/Pillow: 9.4.0.** *Zenodo* 2023**:**doi: 10.5281/zenodo.7498081.

9. Gohlke C: **cgohlke/tifffile: v2023.3.15.** *Zenodo* 2023**:**doi: 10.5281/zenodo.7738996.

10. van der Walt S, Schonberger JL, Nunez-Iglesias J, Boulogne F, Warner JD, Yager N, Gouillart E, Yu T, scikit-image c: **scikit-image: image processing in Python.** *PeerJ* 2014, **2:**e453.

11. Bradski G: **The OpenCV Library. Dr. Dobb's Journal of Software Tools.** *Dr Dobb's J Softw Tools* 2000, **120:**122-125.

12. Bankhead P, Loughrey MB, Fernandez JA, Dombrowski Y, McArt DG, Dunne PD, McQuaid S, Gray RT, Murray LJ, Coleman HG, et al: **QuPath: Open source software for digital pathology image analysis.** *Sci Rep* 2017, **7:**16878.

13. Pebesma E, Bivand R: *Spatial Data Science: With Applications in R.* 1 edn: Chapman and Hall/CRC; 2023.

14. Arganda-Carreras I, Sorzano COS, Marabini R, Carazo JM, Ortiz-de-Solorzano C, Kybic J: **Consistent and Elastic Registration of Histological Sections Using Vector-Spline Regularization.** In *Computer Vision Approaches to Medical Image Analysis CVAMIA 2006 Lecture Notes in Computer Science. Volume* 4241. Edited by Beichel RR, Sonka M. Berlin, Heidelberg: Springer; 2006

15. Ester M, Kriegel H-P, Sander J, Xu X: **A density-based algorithm for discovering clusters in largespatial databases with noise.** *Proc 2nd Int Conf on Knowledge Discovery and Data Mining Portland, OR* 1996**:**226–231.

16. Virtanen P, Gommers R, Oliphant TE, Haberland M, Reddy T, Cournapeau D, Burovski E, Peterson P, Weckesser W, Bright J, et al: **SciPy 1.0: fundamental algorithms for scientific computing in Python.** *Nat Methods* 2020, **17:**261-272.
