# Supplementary material for: Comparison of spatial transcriptomics technologies using tumor cryosections
Source: Genome Biol. 2025 Jun 20;26:176. doi: 10.1186/s13059-025-03624-4 (PMC12180266; doi:10.1186/s13059-025-03624-4)
Supplement: Supplementary file 18 — Additional file 18: Table S10. Data analysis software from this study. [file 13059_2025_3624_MOESM18_ESM.pdf]

**Table S10. Custom data analysis software from this study.**

| Software | Link | Description |
|---|---|---|
| spatial_qc | https://github.com/scOpenLab/spatial_analysis/releases/tag/v1.0.0 | R scripts for QC of ST data. |
| spatial_analysis | https://github.com/scOpenLab/spatial_analysis/releases/tag/v1.0.0 | R-scripts and Jupyter notebooks for analysis of ST data. |
| resolve_processing | https://github.com/scOpenLab/spatial_analysis/releases/tag/v1.0.0 | Tools and scripts for analyzing MC data with a Nextflow pipeline for segmentation and computing transcripts counts per cell |
| seurat_resolve_importer | https://github.com/scOpenLab/spatial_analysis/releases/tag/v1.0.0 | Tools and scripts for analyzing MC data with Seurat. |
| geojson_seurat_cropper | https://github.com/scOpenLab/spatial_analysis/releases/tag/v1.0.0 | Crops a Seurat object using a sf polygon to retain only cells and molecules inside the polygon. |
| xenium_processing | https://github.com/scOpenLab/spatial_analysis/releases/tag/v1.0.0 | Scripts for processing of Xenium data. |
| R scripts for reimaging and image analysis | https://github.com/RippeLab/MBEN/releases/tag/1.0.0 | Scripts used for image preprocessing and analysis. |
