# Supplementary material for: Comparison of spatial transcriptomics technologies using tumor cryosections
Source: Genome Biol. 2025 Jun 20;26:176. doi: 10.1186/s13059-025-03624-4 (PMC12180266; doi:10.1186/s13059-025-03624-4)
Supplement: Supplementary file 12 — Additional file 12: Fig. S8. Clustering and cell type annotation of reimaged Xenium slides. [file 13059_2025_3624_MOESM12_ESM.pdf]

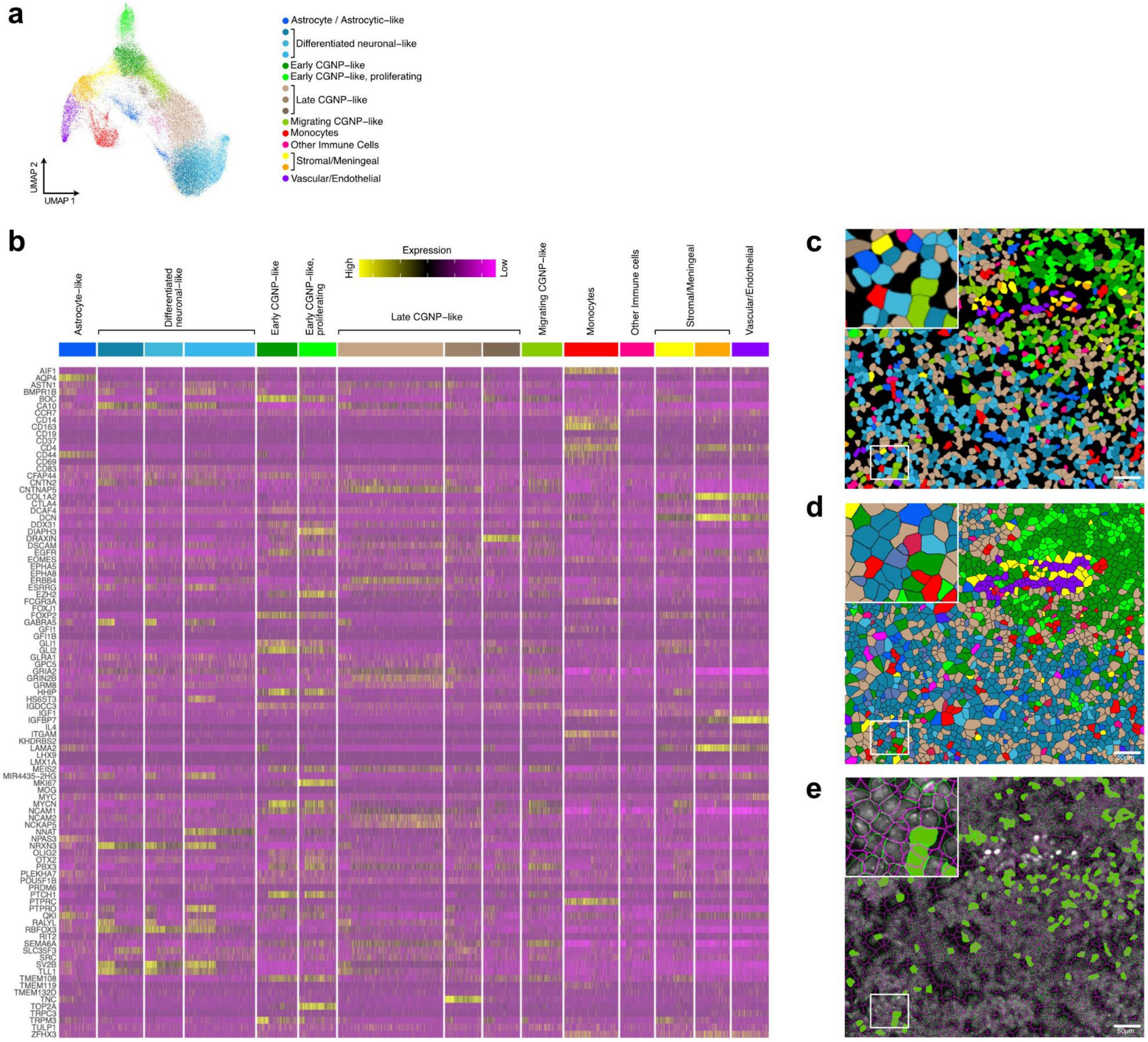

Fig. S8. Clustering and cell type annotation of reimaged Xenium slides

Clustering was conducted on data from samples MB266 and MB295 reimaged by SDCM, which included a total of 52,270 cells. (**a**) Clustering and UMAP visualization of cell-type annotations. (**b**) Gene expression heatmap of the clusters in panel **a**. (**c**) Tissue map of sample MB295 with cells segmented from SDCM reimaging, colored according to their cluster. The zoom-in displays an area rich in migrating cerebellar granule neuronal precursor (CGNP)-like cells. (**d**) Same as panel **c**, but with the original Xenium cell segmentation with expansion and the resulting cell type assignment. (**e**) Image highlighting the difference between the SDCM-based segmentation (green, panel **c**) and the original widefield-based segmentation (magenta, panel **d**). Green cells are migrating CGNP-like cells that could only be detected in the refined SDCM-based segmentation and cell annotation. Scale bars: 50 µm, zoom-ins: 3x magnification.
